# Text Sequence Stimulation for High-Speed and Comfortable SSVEP-BCI

**DOI:** 10.34133/cbsystems.0612

**Published:** 2026-06-15

**Authors:** Xiaoyang Li, Shaojie Zhang, Yonghao Song, Shangen Zhang, Xiaogang Chen, Yijun Wang, Xiaorong Gao

**Affiliations:** ^1^ School of Biomedical Engineering, Tsinghua University, Beijing 100084, China.; ^2^ Tianjin Key Laboratory of Bioelectromagnetic and Intelligent Health, School of Health Sciences and Biomedical Engineering, Hebei University of Technology, Tianjin 300131,China.; ^3^ School of Computer and Communication Engineering, University of Science and Technology Beijing, Beijing 100083, China.; ^4^ Institute of Biomedical Engineering, Chinese Academy of Medical Sciences and Peking Union Medical College, Tianjin 300192, China.; ^5^ Laboratory of Solid-State Optoelectronics Information Technology, Institute of Semiconductors, Chinese Academy of Sciences, Beijing 100083, China.

## Abstract

Steady-state visual evoked potential brain–computer interfaces offer a high-speed communication channel. However, traditional steady-state visual evoked potential paradigms often rely on strong flickering visual stimulation, which can lead to substantial visual fatigue. Moreover, the electroencephalography responses evoked by brightness flicker are spatially constrained and are primarily associated with occipital visual processing. This study presents a novel text sequence stimulation paradigm that combines periodic visual stimulation with orthographic information and elicits distinct occipital and occipitotemporal scalp response patterns relative to conventional brightness flicker. Frequency-sweep experiments were conducted to investigate the temporal, spatial, and spectral characteristics of the evoked responses. A comparison experiment further showed that text sequence stimulation is less sensitive to variations in stimulus size and luminance than conventional brightness flicker. Based on these findings, a 40-target speller was developed and validated through online experiments. The proposed paradigm achieved an information transfer rate of 235.12 ± 30.12 bits/min while significantly improving user comfort, as confirmed by questionnaire evaluations. These results suggest that text sequence stimulation offers a practical design direction for high-speed and more comfortable visual brain–computer interface.

## Introduction

A brain–computer interface (BCI) establishes a direct communication pathway between the brain and external devices, enabling users to perform tasks using neural signals alone [1,2]. Among various BCI paradigms, steady-state visual evoked potential (SSVEP) BCIs have received extensive attention due to their noninvasiveness, high signal-to-noise ratio (SNR), and high information transfer rate (ITR) [3]. With the development of optimized stimuli and decoding algorithm, state-of-the-art SSVEP-based systems have achieved ITR exceeding 400 bits/min [4]. Moreover, SSVEP-BCIs have been applied in practical tasks such as spelling, robotic control, and smart home interaction [5–9].

Despite these advantages, the long-term usability of SSVEP-BCI remains limited by the discomfort caused by visually intensive stimulation. Conventional SSVEP paradigms typically rely on high-contrast brightness flicker to elicit strong neural responses. Such stimuli are known to cause visual fatigue, particularly under prolonged exposure [10]. Fatigue not only degrades user experience but also reduces the amplitude and consistency of the evoked potentials, ultimately impairing system performance [11]. To mitigate this issue, previous studies have explored strategies such as increasing stimulation frequency [12,13], reducing stimulus contrast and area [14–17], and employing colored stimuli [18]. Although these approaches can reduce discomfort, they do not fundamentally resolve the limitations of conventional SSVEP paradigms.

These limitations arise because conventional designs rely primarily on responses associated with occipital visual processing. Reducing flicker intensity often leads to a drop in SSVEP response and classification accuracy. To overcome this trade-off, recent research has explored alternative stimulus designs associated with dorsal stream visual processing, particularly motion-based paradigms [11,19–21]. Steady-state motion visual evoked potential paradigms can improve comfort by reducing salient flicker, but they often yield lower performance because the evoked responses typically show smaller amplitudes and fewer harmonics than conventional brightness-based SSVEP [20].

In contrast, ventral stream-related visual processing remains underexplored in SSVEP-based BCI. The ventral stream plays an important role in face, object, and orthographic processing and is known to exhibit a degree of response invariance [22–29]. Promising results have also been reported in P300-based BCI using semantically rich stimuli associated with ventral stream processing [30,31]. Among the stimulus classes associated with ventral stream, text may offer unique advantages. Intracranial recordings have shown broad ventral activation in response to text stimuli, while frequency-tagged electroencephalography (EEG) studies have shown that steady-state responses to written words exhibit distinct spatial response patterns over occipitotemporal regions [32,33]. Together, these findings suggest that text stimuli may evoke brain responses in a way that differs from conventional brightness flicker. In addition, text stimuli can be implemented with a small visual area and low luminance variability, and repeated presentation has been reported to evoke stronger neural responses than faces or symbols [34].

In this study, we propose a novel text sequence stimulation paradigm for SSVEP-based BCI. We hypothesize that such stimuli can evoke robust SSVEP responses by combining periodic visual stimulus with orthographic information. Compared with conventional SSVEP paradigms, orthographic processing is often more tolerant to variations in size and luminance, which may make text stimuli particularly suitable for smaller and more visually gentle stimulation designs. To evaluate this approach, we first conducted a frequency-sweep experiment to characterize the EEG responses elicited by text stimuli. We then compared the neural responses to text and brightness stimulation under different size and luminance conditions. Finally, we developed a 40-target speller based on text sequence stimulation and evaluated its performance through online spelling tasks.

## Materials and Methods

### Subjects

A total of 21 subjects (9 females, aged 20 to 33 years, mean age 26.62) participated in the frequency-sweep experiment, 15 subjects (7 females, aged 21 to 33 years, mean age 25.33) participated in the comparison experiment evaluating the effects of stimulus luminance and size between text and brightness stimuli, and 14 subjects (7 females, aged 23 to 36 years, mean age 27.29) participated in the online experiment. Among them, 2 subjects in the frequency-sweep experiment had no prior experience with BCI, whereas all participants in the comparison and online experiment had previous BCI experience. Importantly, all subjects were using the text sequence stimulation paradigm for the first time. All participants were native Mandarin speakers with normal or corrected-to-normal vision. The study protocol was approved by the Ethics Committee of Tsinghua University, and written informed consent was obtained from each participant prior to the experiment.

### Visual stimulus presentation

Stimuli were displayed on a 24.5-inch liquid crystal display monitor (Dell AW2518H) with a screen resolution of 1,920 × 1,080 pixels and a refresh rate of 240 Hz. The stimulus presentation program was developed using the Psychophysics Toolbox (PTB-3) in MATLAB 2015b.

The stimulus material consisted of commonly used 100 Chinese characters with an average stroke count of 5.94. The characters were displayed in the commonly used “Simsun” font presented in 100 text size with white text on a black background in the frequency-sweep experiment and online experiment. In the comparison experiment, 3 stimulus sizes (50, 100, and 150) and 3 luminance levels—low (30), medium (127), and high (255)—were used to evaluate the effects of stimulus luminance and size.

Text stimuli significantly reduced the visual stimulation area compared to conventional brightness flicker stimuli, occupying only approximately 14% of their area when presented in white font. Specifically, in the text sequence stimulation paradigm, the luminance of individual characters ranged from 16.5 to 143.8 cd/m^2^, with an average luminance of 100.6 cd/m^2^. In contrast, the luminance of conventional SSVEP flicker stimuli varied from 0 to 423 cd/m^2^, indicating a substantially higher dynamic luminance range.

**Fig. 1.**
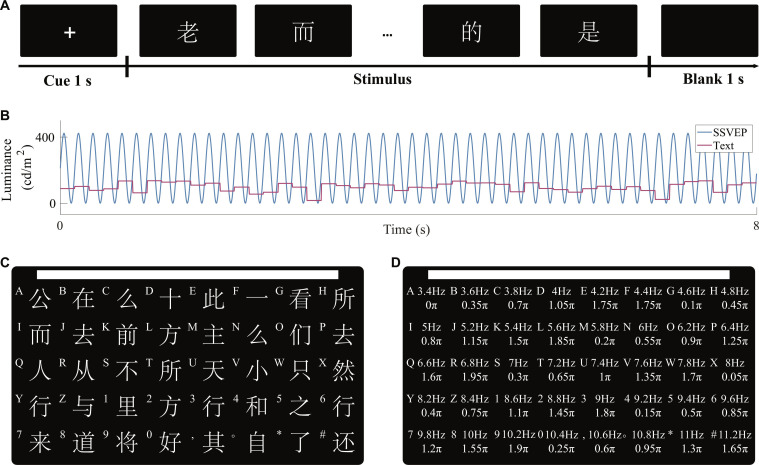
Experimental paradigm for text sequence stimulation. (A) Schematic of a single-trial stimulus process in the frequency-sweep experiment and the comparison experiment. (B) Comparison of luminance changes between text sequence stimulation and brightness-modulated steady-state visual evoked potential (SSVEP) under 6 Hz. (C) Interface of the 40-target brain–computer interface speller. (D) Frequency and phase information for each target in the 40-target brain–computer interface using joint frequency-phase modulation (JFPM) encoding method.

In the frequency-sweep and comparison experiments, Chinese characters were presented sequentially at different frequencies during the stimulation process, with each character appearing random with equal probability, as shown in Fig. 1A. In the online experiment, stimuli were encoded using joint frequency-phase modulation (JFPM) method [3], where all targets changed simultaneously with different frequency-phase parameters. Each visual flicker was presented on the screen based on the square wave signal variation corresponding to the respective frequency and phase. The characters changed at each rising edge of the square wave signal as Eq. (1):

$$s\left(f,\;\varphi ,\;g\right)=\text{square}\left(\frac{2\pi \mathrm{fg}}{\text{freshrate}}+\varphi \right)$$ (1)

where f represents the stimulus frequency, φ denotes the initial phase of the stimulus, and g indicates the index of the refresh frame since the start of the stimulation.

In detail, single-target frequency-sweep experiments were conducted with stimulus frequencies ranging from 3 to 12 Hz in 0.2-Hz increments, with the stimulus phase set to 0. Fig. 1B illustrates the luminance comparison between the 2 paradigms at 6 Hz, showing that text stimulation produced smaller luminance changes than brightness-based flicker. For the comparison experiments, 4 stimulation frequencies (3, 6, 9, and 12 Hz) were employed, also with the phase set to 0. For the online experiments, a JFPM encoding method was adopted, in which all targets changed according to their specific frequency-phase sequences. The 40-target speller (Fig. 1C and D) used stimulation frequencies from 3.4 to 11.2 Hz with a 0.2-Hz interval, combined with a phase interval of 0.35π.

### Experimental procedure

The frequency-sweep experiment consisted of 12 blocks, with each block comprising 46 trials corresponding to 46 different stimulus frequencies. Each trial followed the sequence shown in Fig. 1A, consisting of 1 s of fixation cross, 8 s of text sequence stimulation, and 1 s of black screen. The next trial commenced immediately after the black screen. The detailed experimental procedure is illustrated in Fig. S1A.

The comparison experiment consisted of 8 blocks, each containing stimuli at 4 frequencies (3, 6, 9, and 12 Hz) combined with 9 size-luminance conditions. Each trial consisted of 1 s of fixation cross, 5 s of text sequence stimulation, and 1 s of black screen. The detailed experimental procedure is illustrated in Fig. S1B.

The online experiment employed a cue-guided spelling task, comprising 20 blocks. The first 12 blocks were designated for data collection, while the subsequent 8 blocks were used for performance testing. Each block consisted of 40 trials, and each trial began with a random cue lasting 0.5 s. Following the cue, all targets flickered for 0.7 s, a duration determined based on the offline results where 90% classification accuracy was achieved [35]. Subjects fixated on the Chinese characters changing at the cued location during this process. After the stimulus presentation, the results were presented to the subjects in real time. The detailed experimental procedure is illustrated in Fig. S1C.

### Data recording

During the experiment, subjects were seated quietly in a well-lit indoor environment, approximately 70 cm away from the screen [36]. The Synamp2 system (Neuroscan, Inc.) was utilized to collect EEG data at a sampling rate of 1,000 Hz, with the ground electrode placed at AFz and the reference electrode at the vertex. In both experiments, EEG data were recorded using full-head electrode arrays in Quik-Cap 64 according to the 10-20 system. We defined 3 preselected scalp electrode subsets for channel-ablation analysis: occipital (Oz, O1, and O2), occipitotemporal (T7, T8, TP7, TP8, P7, P8, PO7, PO8, Oz, O1, and O2), and parietal (Oz, POz, Pz, Cpz, and Cz) subsets [23].

### Data analysis

In this study, due to the inherent delay in the visual system, we uniformly extracted EEG data from 0.14 to 5.14 s for analysis, with 140 ms accounting for visual latency [3]. The extracted data was downsampled to 250 Hz and filtered using a notch filter to remove the 50-Hz power line interference, followed by a fourth-order Butterworth band-pass filter with a range from 2 to 60 Hz. EEG signals were then re-referenced using common average referencing, and independent component analysis was applied to remove ocular artifacts. SSVEP response was assessed using narrowband SNR [37]. The SNR was computed as the ratio of the spectral amplitude of the signal to the mean spectral amplitude in the nearby frequencies, as described by the following Eq. (2). Specifically, the EEG data were first averaged across trials, and the fast Fourier transform (FFT) was then computed on the averaged waveform.

$$\mathrm{SNR}\left(f\right)=20\times \log_{10}\left(\frac{r\cdot Y\left(f\right)}{\sum_{m=1}^{r/2}\left[Y\left(f-0.2m\right)+Y\left(f+0.2m\right)\right]}\right)$$ (2)

where f represents the signal frequency, $Y\left(•\right)$ denotes the amplitude spectrum calculated by FFT, and m is an index for summation ranges from 1 to r/2. In this study, r is set to 10. With data duration of 5 s, the frequency resolution is 0.2 Hz. When r is set to 10, it reflects the ratio of the signal to the EEG background activity within the surrounding ±1-Hz range. The scalp SNR was computed using the average of the posterior 39 channels used for classification (Cz, C1, C2, C3, C4, C5, C6, T7, T8, CPz, CP1, CP2, CP3, CP4, CP5, CP6, TP7, TP8, Pz, P1, P2, P3, P4, P5, P6, P7, P8, POz, PO3, PO4, PO5, PO6, PO7, PO8, Cb1, Oz, O1, O2, and Cb2). These posterior channels cover occipital, occipitotemporal, and parietal scalp, providing a broad representation of posterior visual responses.

### Decoding algorithms

In the decoding process, raw EEG data were used without applying common average referencing and independent component analysis procedures. In an offline experiment, we compared the EEG data decoding performance between the task-related component analysis (TRCA) and task-discriminant component analysis (TDCA) algorithms [38,39]. The TDCA algorithm exhibited higher accuracy and thus was employed for EEG decoding in the online experiments. The TRCA algorithm computes spatial filters $w_{i}$ separately for each stimulus target to extract task-relevant components by maximizing intertrial reproducibility. The calculation of spatial filter is shown in Eq. (3).

$$\text{maximiz}e_{w_{i}}=\frac{w_{i}^{T}Sw_{i}}{w_{i}^{T}Qw_{i}}$$ (3)

$$\begin{array}{c} w_{i}^{T}Qw_{i}=\sum_{\begin{array}{c} h1,h2=1\\ h1\neq h2 \end{array}}^{N_{t}}\sum_{j1,j2=1}^{N\mathrm{ch}}w_{j1}w_{j2}\mathrm{Cov}\left(x_{j1}^{\left(h1\right)}\left(t\right),\;x_{j2}^{\left(h2\right)}\left(t\right)\right) \end{array}$$ (4)

$$w_{i}^{T}Sw_{i}=\sum_{j1,j2=1}^{N\mathrm{ch}}w_{j1}w_{j2}\mathrm{Cov}\left(x_{j1}\left(t\right),\;x_{j2}\left(t\right)\right)$$ (5)

$w_{i}^{T}Qw_{i}$ and $w_{i}^{T}Sw_{i}$ are shown in Eqs. (4) and (5); j is the index of channels, $N_{\mathrm{ch}}$ is the number of channels, h is the index of trials, $N_{t}$ is the number of trials, $x_{j}^{h}$ represents EEG data from *j*-th channels and *h*-th trials, and $\mathrm{Cov}\left(•\right)$ calculates the covariance. An ensemble method is used to generate a global spatial filter $W_{\text{TRCA}}=\left[w_{1}.\ldots w_{i}..\ldots w_{N_{c}}\right]$, where $N_{c}$ denotes the number of classes, and $N_{c}=40$ in this study.

TDCA algorithm generates a spatial filter, denoted as W, for all stimulus targets simultaneously. Based on the Fisher criterion, it maximizes interclass clustering while minimizing intraclass distance. Consequently, TDCA enhances the discriminability between different classes and improves the SSVEP decoding performance. The TDCA algorithm also incorporates data delay augmentation. $\tilde{X}$ denotes the augmented EEG trial, where lag represents the temporal delay length and is defined as a positive integer.

$$\tilde{X}=\left[X^{\text{T}},\;X_{1}^{T},\ldots ,\;X_{\mathrm{lag}}^{T}\right]$$ (6)

The within-class $H_{w}$ and between-class distributions $H_{b}$ are shown in Eqs. (7) and (8), respectively.

$$H_{w}=\frac{1}{\sqrt{N_{t}}}\left[X^{\left(1\right)}-{\bar{X}}^{\left(1\right)},\;\ldots X^{\left(N_{t}\right)}-{\bar{X}}^{\left(N_{t}\right)}\right]$$ (7)

$$H_{b}=\frac{1}{\sqrt{N_{c}}}\left[{\bar{X}}^{1}-{\bar{X}}^{\mathrm{all}},\;\ldots {\bar{X}}^{N_{c}}-{\bar{X}}^{\mathrm{all}}\right]$$ (8)

${\bar{X}}^{i}$ and ${\bar{X}}^{\left(i\right)}$ denote the 2 dimensional centers of i-th class and i−th sample after augmentation. The calculation of spatial filter is shown in Eq. (9).

$${\text{maximize}}_{w}=\frac{\mathrm{tr}\left(W^{T}S_{b}W\right)}{\mathrm{tr}\left(W^{T}S_{w}W\right)}$$ (9)

In Eq. (9), $S_{b}=H_{b}{H_{b}}^{T}$, $S_{w}=H_{w}{H_{w}}^{T}$. The EEG data were projected into multiple subspaces using the spatial filter matrix $W=\left[w_{1}.\ldots w_{i}..\ldots w_{N_{k}}\right]$, and subspace selection was employed to retain an optimal number of components, thereby enhancing classification performance. In this experiment, the TDCA algorithm’s subspace $N_{k}$ and delay lag were set to 6 and 4, respectively.

### Recognition procedure

The offline and online decoding procedures were based on the same subject-specific TDCA framework. The detailed decoding procedure of the proposed system is shown in Fig. S2. For the TRCA algorithm, the same decoding framework was used, with the TDCA projection step replaced by TRCA projection. During decoding, online EEG data were segmented according to the trigger signals. To ensure that phase intervals were consistent with those used in the online experiment and to better evaluate classification performance using the offline data, the offline EEG data were phase-shifted to simulate the phase encoding used online, as illustrated in Fig. S3. Line-noise interference was then removed before further processing.

The preprocessed signals were then decomposed into multiple sub-bands using a filter bank, as summarized in Table S1. For each sub-band, the data were projected into the TDCA subspace and matched with the corresponding template to obtain a correlation coefficient. The coefficients from different sub-bands were subsequently combined using the weighted fusion method, with the weights of different sub-bands given by Eq. (10), and the target with the maximum score was taken as the final recognition result.

$$w\left(n\right)=n^{-a}+b$$ (10)

Based on the offline experimental results, the online experiments adopted a stimulus duration of 0.7 s, 39 channels for classification, 5 sub-bands, and weighting coefficients of *a* = 2 and *b* = 0.1. The detailed parameter optimization process is illustrated in Fig. S4. Except for the analyses explicitly examining different data lengths, a fixed data length of 0.7 s was used throughout the study. For offline analysis, the subject-specific TDCA model was obtained using a leave-one-trial-out cross-validation scheme, with 11 trials for training and 1 trial for testing. For online analysis, the subject-specific TDCA model was derived from the training data and then applied to real-time decoding using the same pipeline.

### Performance evaluation

To evaluate the decoding performance of the BCI system, we employed ITR as an evaluation metric. The formula for calculating ITR is shown in Eq. (11).

$$\mathrm{ITR}=\frac{60}{T}\cdot \left(\log_{2}N_{c}+P\cdot \log_{2}P+\left(1-P\right)\cdot \log_{2}\frac{1-P}{N_{c}-1}\right)$$ (11)

In the equation, $N_{c}$ represents the number of targets in the BCI system, which in this experiment is 40. *P* represents the classification accuracy of the system. *T* denotes the system’s recognition time, which in this study is the data time used for classification plus 0.5 s of gaze shift time.

### Questionnaire evaluation

We evaluated the comfort of the proposed text sequence BCI paradigm (Fig. 1C) and compared it with a conventional SSVEP-BCI [39] using 2 questionnaires. First, a comfort questionnaire was employed. The questionnaire included the following aspects: (a) preference for stimulus mode (rated from 1: very likable to 5: very unlikable); (b) stimulus comfort level (rated from 1: very comfortable to 5: very uncomfortable); and (c) perception of stimulus flicker (rated from 1: hardly noticeable to 5: very noticeable) [13]. Second, to obtain a more comprehensive evaluation, the NASA Task Load Index (NASA-TLX) was administered to assess subjective workload. The NASA-TLX includes 6 subscales: mental demand, physical demand, temporal demand, performance, effort, and frustration, each rated on a 0 to 100 scale [19]. All participants in the online experiment completed the questionnaires. During the evaluation session, subjects were randomly assigned to observe either the text sequence BCI or the conventional SSVEP-BCI first, with each paradigm presented for 5 min. After exposure, participants rated the paradigm using the 2 questionnaires.

To better demonstrate the advantages of low-frequency stimulation and evaluate comfort across the usable frequency range, an additional comfort questionnaire evaluation experiment was conducted. Subjects were asked to observe brightness flicker stimuli and text sequence stimuli at frequencies ranging from 1 to 20 Hz and rate the different frequencies under each stimulus mode. Different frequencies of text stimuli and brightness flicker stimuli were randomly distributed across 40 positions on the screen and flashed simultaneously.

## Results

### Text sequence response pattern

**Fig. 2.**
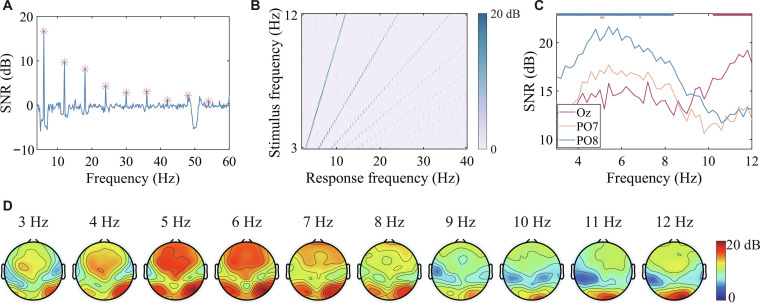
Frequency domain response characteristics of text sequence stimulation. (A) Average signal-to-noise ratio (SNR) spectrum at 6-Hz stimulation averaged across all subjects. (B) The relationship between stimulation frequency and electroencephalography (EEG) SNR in the range of 3 to 12 Hz. (C) Fundamental frequency SNR variations with stimulation frequency for PO7/8 and Oz. The blue line at the top indicates significantly higher responses in the right occipitotemporal region compared to the occipital region. The light orange line indicates significantly higher responses in the left occipitotemporal region compared to the occipital region. The red line in the high-frequency range indicates significantly higher responses in the occipital region compared to bilateral occipitotemporal region (paired *t* test, *P* < 0.05). (D) Average SNR topographical maps of text sequence stimulation.

FFT analysis reveals significant EEG responses at the frequency of text presentation and its harmonics. Fig. 2A depicts the SNR of EEG responses at 6-Hz stimulation, where the fundamental frequency shows the highest SNR, and the SNR of harmonic responses gradually decreases. Fig. 2B illustrates the relationship between stimulation frequency and response SNR, indicating the presence of fundamental and multiple harmonic responses across all 46 stimulation frequencies. As shown in Fig. 2C, the SNR of EEG responses at different frequencies was compared between the left occipitotemporal (PO7), right occipitotemporal (PO8), and occipital (Oz) regions. Within the 3- to 8.4-Hz range, the right temporoparietal region exhibited the strongest EEG response. At 5, 5.2, and 6.8 Hz, the response in the left occipitotemporal region was significantly stronger than that in the occipital area. Beyond 10.2 Hz, the response in the occipital region gradually strengthened, showing a significantly higher SNR than bilateral occipitotemporal regions.

As shown in Fig. 2D and Fig. S5, 2 distinct scalp response patterns to text stimulation were evident within the 3- to 12-Hz range. Between 3 and 8 Hz, responses were predominantly localized over bilateral occipitotemporal scalp regions. As the frequency increased to around 9 to 10 Hz, the response pattern shifted from an occipitotemporal distribution toward occipital dominance, suggesting a frequency-dependent change in scalp response distribution. At 10 to 12 Hz, the EEG response patterns elicited by text stimulation became more similar to those elicited by brightness flicker stimulation in Fig. 4B.

### Time course of text response

**Fig. 3.**
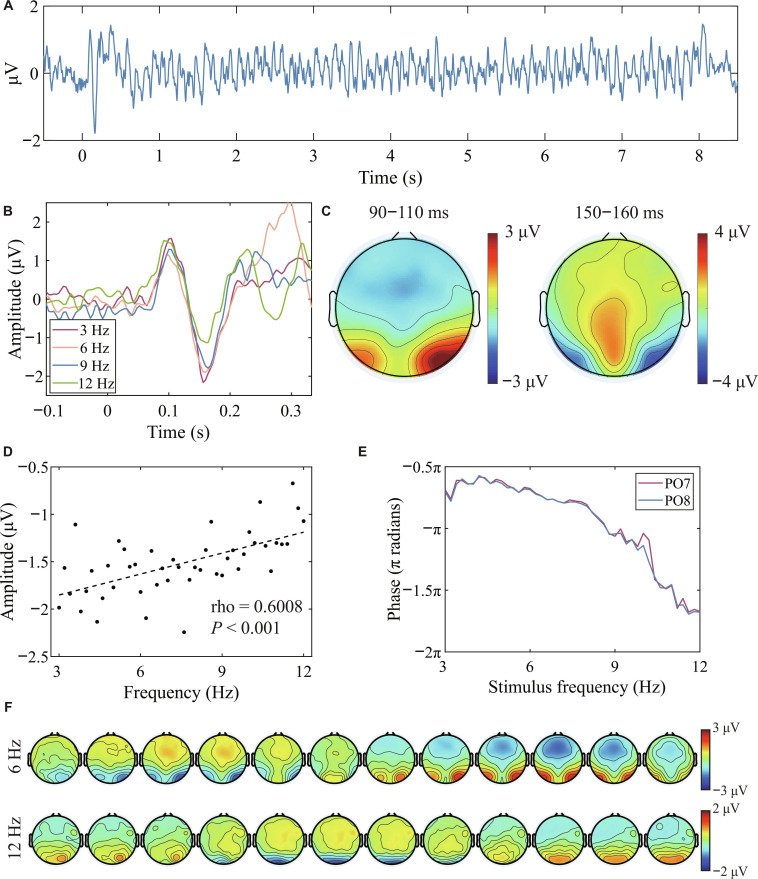
Temporal response characteristics of text stimulation averaged across all subjects. (A) Temporal response waveforms within −0.5 to 8.5 s under 9-Hz stimulation. (B) Transient response waveforms under 3-, 6-, 9-, and 12-Hz stimulation. (C) P1 and N170 response topographies under 12-Hz stimulation. (D) Relationship between stimulation frequency and N170 amplitude. (E) Response phases of PO7 and PO8 channels under 3- to 12-Hz stimulation. (F) Response topographies within a cycle under 6- and 12-Hz stimulation, with each topography representing the average response within a 30° phase.

The temporal dynamics of text sequence responses, derived from the same 39 channels used for SNR calculations, are depicted in Fig. 3. For EEG responses under 9-Hz stimulation, as shown in Fig. 3A, there is a clear distinction between transient and steady-state responses following the onset of stimulation. The transient response time course for different stimulation frequencies, as shown in Fig. 3B, reveals a positive peak around 100 ms (P1) and a negative wave around 155 ms (N170). The response topography of P1 and N170 under 12-Hz stimulation is illustrated in Fig. 3C. Both P1 and N170 predominantly reflect responses in the occipitotemporal regions. Transient topographies across all stimulation frequencies show similar response patterns. Although the response pattern remains consistent, the amplitude of N170 is affected by subsequent stimuli. As shown in Fig. 3D, the N170 amplitude gradually decreases with increasing stimulation frequency, indicating that the transient response is influenced by subsequent stimulus masking.

During the steady-state process, phase changes in responses of the left and right occipitotemporal regions are shown in Fig. 3E. Under the occipitotemporal response pattern, while there are significant differences in response SNR between the left and right sides, the phase remains consistent. The topographical changes during the steady-state process at 6- and 12-Hz stimulation are shown in Fig. 3F. At 6-Hz stimulation, differences in amplitude between the left and right occipitotemporal regions are observed, but the temporal dynamics are relatively consistent. Under 12-Hz stimulation, periodic EEG activity predominantly originating from the occipital lobe was observed. The varying spatiotemporal response patterns present greater challenges for classifier design.

### Effects of size and luminance variations

Occipital visual responses are highly sensitive to stimulus size and luminance [40], whereas orthographic processing is often more tolerant to such variations [26,41]. To examine whether the proposed text sequence stimulation paradigm and conventional brightness stimulation differ in this respect, we manipulated both stimulus size (50, 100, and 150) and luminance (30, 127, and 255).

**Fig. 4.**
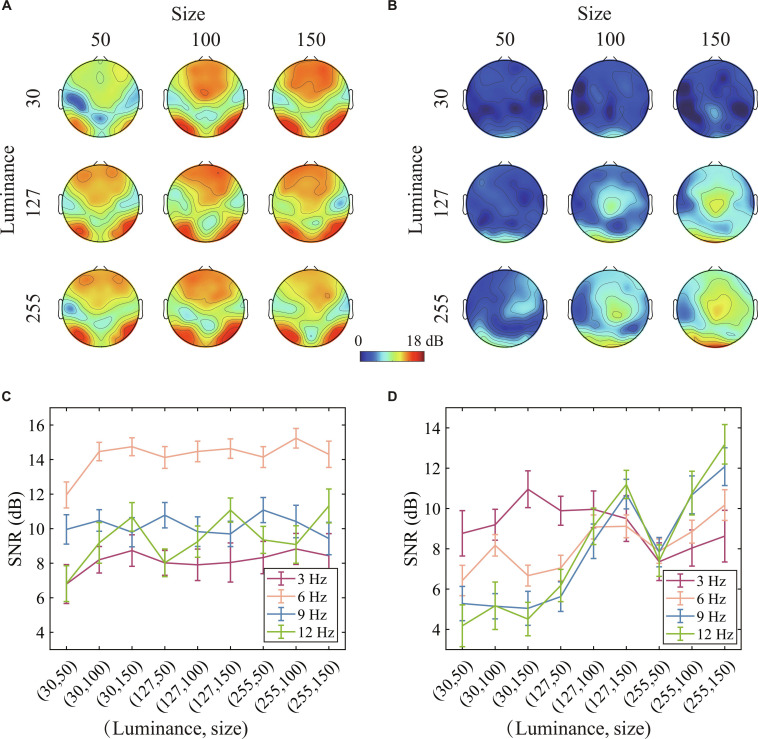
Comparison of text and conventional brightness stimulation responses across different stimulus sizes and luminance. (A) Topographies of 6-Hz text stimulation across different stimulus conditions. (B) Topographies of 12-Hz brightness stimulation across different stimulus conditions. (C) Signal-to-noise ratio (SNR) of text stimulation at 3, 6, 9, and 12 Hz across different stimulus conditions. (D) SNR of brightness stimulation at 3, 6, 9, and 12 Hz across different stimulus conditions. Error bars indicate standard error across subjects.

Under size-luminance manipulation, the 2 paradigms showed distinct response trends, as shown in Fig. 4. At 6 Hz, text sequence stimulation elicited relatively stable occipitotemporal activation across conditions, with limited dependence on stimulus size or luminance. By contrast, at 12 Hz, brightness flicker stimulation produced more occipital-dominant responses, and the response strength increased with stimulus size and luminance. The corresponding SNR results were consistent with these topographic patterns, indicating that brightness flicker stimulation was more sensitive to physical stimulus parameters, whereas text sequence stimulation remained comparatively stable across most tested conditions.

In Fig. 4C and D, we further compared the 2 stimulation paradigms. For the text paradigm, 2-way repeated-measures analyses of variance (ANOVAs) revealed no significant main effects of stimulus size or luminance at 3 and 9 Hz. At 6 Hz, responses were also largely invariant across conditions, with no significant main effects of stimulus size or luminance in the smallest and dimmest condition. At 12 Hz, however, text-evoked responses were significantly modulated by size (*F* = 13.17, *P* < 0.001). Across all frequencies, the variability of text-evoked SNR remained modest (2.03, 3.28, 1.65, and 4.50 dB at 3, 6, 9, and 12 Hz, respectively).

In contrast, the brightness paradigm exhibited strong dependence on both size and luminance. At 3 Hz, SNRs were significantly modulated by luminance (*F* = 5.45, *P* < 0.05), and at 6 Hz, by size (*F* = 5.62, *P* < 0.01). At 9 Hz, significant main effects of luminance (*F* = 25.36) and size (*F* = 27.16), as well as their interaction (*F* = 6.09), were observed (all *P* < 0.001). At 12 Hz, significant main effects of luminance (*F* = 30.25) and size (*F* = 22.53), together with a size-luminance interaction (*F* = 8.88), were observed (all *P* < 0.001). The variability of brightness-evoked SNRs was substantially larger (3.40, 3.74, 7.03, and 9.00 dB across 3, 6, 9, and 12 Hz, respectively) compared to the text paradigm.

Together, these results indicate that the proposed text sequence stimulation paradigm was less sensitive to variations in stimulus size and luminance than conventional brightness stimulation. At the same time, the comparison in the present study was made at the paradigm level, where multiple stimulus properties jointly shaped the evoked responses. Therefore, the present results suggest that luminance variation alone is insufficient to explain the observed differences, although the experiment was not designed to isolate a single causal factor. To further examine whether factors beyond luminance variation contributed to these differences, we included an additional control experiment in the Supplementary Materials comparing intact common characters with ratio-controlled scrambled-character stimuli. The results showed that preserving character structure was associated with distinct steady-state response characteristics (Fig. S6 and S7).

### Performance evaluation

**Fig. 5.**
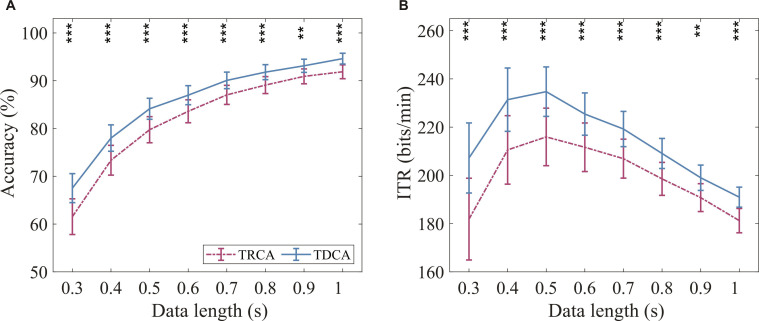
Performance of the 40-target text-based brain–computer interface (BCI) paradigm at different time lengths in frequency-sweep experiment averaged across all subjects. (A) Classification accuracy and (B) information transfer rate (ITR) (paired *t* test, **P < 0.01, ***P < 0.001). Error bars indicate standard error across subjects.

Based on the data from the frequency-sweep experiment, we evaluated the system’s classification accuracy and ITR over different time lengths using the TRCA and TDCA algorithms, as shown in Fig. 5. Classification accuracy was evaluated using leave-one-out cross-validation, with training data consisting of 11 trials. A selection of the posterior 39 channels from the electrode cap and 5 sub-bands are utilized. The classification performance of both TRCA and TDCA algorithms improves with increasing data length, with TDCA achieving an average classification accuracy exceeding 90% at a data length of 0.7 s. The ITR initially increases and then decreases with data length, reaching its maximum at a data length of 0.5 s. Comparing the classification accuracy and ITR between the 2 algorithms, TDCA outperforms TRCA across all data lengths (paired *t* test). Notably, the best subject achieved an accuracy of 96.04% and an ITR of 365.43 bits/min at a data length of 0.3 s. This performance is comparable to that of conventional SSVEP-BCI, which achieve a maximum of 376.58 bits/min and an average of 325.33 bits/min [39].

**Fig. 6.**
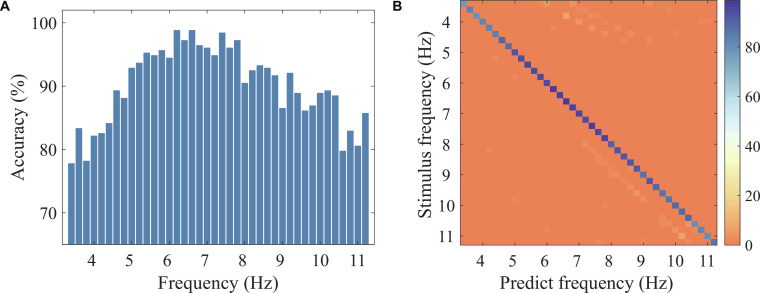
Classification accuracy and confusion matrix at different stimulation frequencies in the offline frequency-sweep experiment averaged across all subjects. (A) Classification accuracy across the 40 target-encoding frequencies selected from the offline frequency-sweep experiment. (B) Confusion matrix across the 40 target-encoding frequencies.

In the frequency-sweep experiment, the confusion matrices between different targets and the classification accuracy under various stimulation frequencies are presented in Fig. 6. The stimulation frequency significantly impacts the classification accuracy. As shown in Fig. 6A, lower classification accuracy is observed in both low- and high-frequency bands, consistent with the SNR distribution. Classification accuracy exceeds 90% in the 5–8.8-Hz and 9.2-Hz stimulation frequency ranges. Fig. 6B reveals that due to harmonic overlap, responses to 3- to 4-Hz stimulation are prone to misclassification into their respective harmonic proximities. High-frequency stimulation tends to result in misclassification into adjacent frequency bands.

### Performance evaluation of different brain regions

**Fig. 7.**
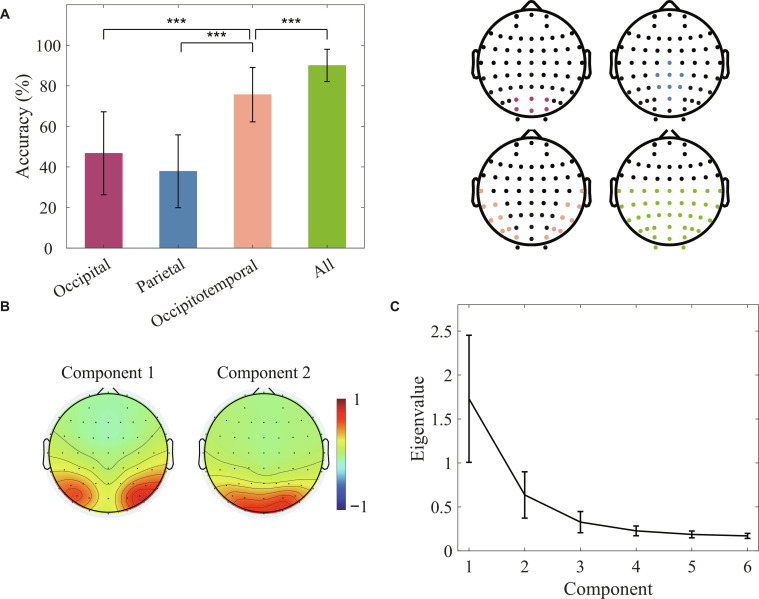
Regional decoding performance and task-discriminant component analysis (TDCA) spatial patterns for the proposed text sequence stimulation paradigm averaged across all subjects. (A) Classification accuracy for predefined scalp electrode subsets (occipital, occipitotemporal, and parietal) and the optimized full-channel configuration (paired *t* test, ***P < 0.001). Specific electrode locations are shown in the right panel. (B) Representative TDCA spatial patterns showing the scalp distribution of discriminative weights. (C) Eigenvalue distribution corresponding to different components. Error bars indicate standard error across subjects.

Visual processing of text is a complex process involving both early occipital responses and higher-level form-related processing, with interactions across these stages [28]. To further examine the spatial distribution of discriminative information, we compared decoding performance across predefined scalp electrode subsets and the optimized full-channel configuration (Fig. 7A). The full-channel configuration achieved the highest classification accuracy, indicating that the proposed text sequence stimulation paradigm benefits from distributed information across posterior scalp regions. Among the regional subsets, the occipitotemporal subset yielded the highest accuracy, followed by the occipital subset, whereas the parietal subset showed the lowest performance.

This regional difference was also reflected in the TDCA spatial patterns shown in Fig. 7B and C. Larger discriminative weights were observed over occipitotemporal and occipital scalp regions, whereas the parietal subset contributed less strongly. Notably, these 2 dominant discriminative patterns correspond well to the 2 major scalp response patterns shown in Fig. 2D, suggesting that the TDCA decoder effectively captured the principal spatial response modes elicited by the proposed paradigm. The lower accuracy of the parietal subset may indicate that the proposed text sequence stimulation paradigm provides relatively less discriminative information over parietal scalp regions, possibly reflecting relatively limited motion- or spatial-attention-related discriminative information over parietal scalp regions.

The occipital subset, although still informative, performed worse than the occipitotemporal subset. This may partly relate to the stimulation frequency setting used in the present study: Most stimulation frequencies elicited stronger occipitotemporal response patterns, whereas more occipital-dominant patterns were mainly observed at the higher-frequency end. As a result, restricting decoding to occipital channels alone may discard part of the discriminative information carried by the occipitotemporal response mode. Overall, Fig. 7 indicates a nonuniform scalp distribution of decoding information under the proposed paradigm, with the most informative signals concentrated over occipital and occipitotemporal regions.

### Online performance

Table 1 displays the performance of 14 subjects in the online experiments. Optimized parameters were used in the online decoding process for all subjects to achieve better overall performance. All participants demonstrated satisfactory performance. The average accuracy of all subjects in the online experiments was 93.71%±6.77%, corresponding to an ITR of 235.12 bits/min ±30.12 bits/min. The best-performing subjects achieved 100% accuracy and an ITR of 266.10 bits/min, while the lowest-performing subject achieved close to 80% accuracy and an ITR of 173.75 bits/min.

**Table 1.** Results of the online experiment

Subject	Accuracy/%	ITR/(bits·min^-1^)
Sub1	91.56	222.93
Sub2	98.13	254.42
Sub3	85.94	199.64
Sub4	99.38	261.71
Sub5	92.81	228.46
Sub6	79.06	173.75
Sub7	97.81	252.72
Sub8	100	266.10
Sub9	82.81	187.58
Sub10	98.13	254.42
Sub11	100	266.10
Sub12	99.06	259.79
Sub13	93.13	229.87
Sub14	94.06	234.16
**Mean ± SD**	**93.71 ± 6.77**	**235.12 ± 30.12**

ITR, information transfer rate

### Questionnaire evaluation

**Fig. 8.**
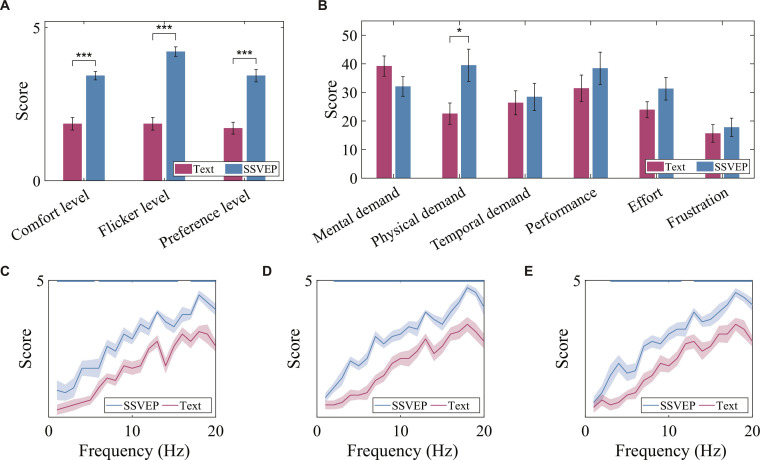
Questionnaire evaluation between text stimulation and brightness stimulation. (A) Subjective comfort evaluation of the 40-target paradigm with text stimulation and the brightness steady-state visual evoked potential (SSVEP) paradigm at 8 to 15.8 Hz. (B) NASA Task Load Index (NASA-TLX) workload ratings for the 2 paradigms. Error bars indicate standard error across subjects. (C) Comfort evaluation of text and brightness stimulation across 1- to 20-Hz frequencies. (D) Flicker perception ratings across 1- to 20-Hz frequencies. (E) Preference ratings across 1- to 20-Hz frequencies. The shaded area represents the standard error across subjects. The top blue lines indicate that the evaluation scores for text stimulation are significantly lower than those for brightness stimulation (paired *t* test, *P < 0.05, ***P < 0.001).

The user experience ratings, as shown in Fig. 8A, indicate that compared with brightness-modulated SSVEP-based BCI paradigms, the text sequence stimulation method demonstrates clear advantages in terms of comfort, flicker perception, and user preference. The NASA-TLX results (Fig. 8B) showed a slight increase in mental demand for text stimulation relative to brightness stimulation, but this difference was not statistically significant (*P* > 0.05). In contrast, physical demand was significantly higher for brightness stimulation, likely due to the need to maintain sustained fixation (*P* < 0.05). For other dimensions of workload—including temporal demand, performance, effort, and frustration—the ratings for text stimulation were generally more favorable than for brightness stimulation, with no significant differences observed.

Both the text sequence method and brightness modulation method exhibit similar trends with increasing frequency (Fig. 8C to E); the scores increase, indicating stronger flicker perception and a corresponding decline in comfort and preference. Except for the extremely low frequency of 1 Hz, the text sequence stimulation consistently outperforms traditional brightness-modulated stimulation in terms of user experience across almost all frequency bands. Thirteen-hertz text stimulation was reported to be less comfortable compared to 8-Hz brightness-modulated stimulation, which represents the lower frequency limit commonly used in brightness modulation paradigms [3,39].

## Discussion

In this study, we proposed a visual BCI paradigm based on text sequence stimulation, achieving both comfortable visual stimulation and high performance. Using stimulus frequencies ranging from 3.4 to 11.2 Hz, we designed a 40-target BCI spelling keyboard. In offline experiments, this paradigm achieved an average ITR of 219.22 bits/min, with a maximum of 365.42 bits/min, and online validation using the TDCA algorithm yielded an average ITR of 235.12 bits/min ± 30.12 bits/min. Compared with conventional brightness flicker paradigms, the brightness changes induced by character transitions more closely resemble those encountered during natural reading, which may partly explain the improved subjective comfort observed here. Questionnaire evaluations also showed significantly higher comfort levels with text sequence stimulation than with the conventional brightness flicker method.

Table 2 summarizes representative subtle flicker SSVEP-BCI paradigms in recent years. The ITR of current subtle flicker BCI systems remains relatively low, with the highest reported value reaching 153.79 bits/min [12], which is lower than the 235.12 bits/min achieved with text sequence stimulation. Most current subtle flicker BCI systems still rely on brightness modulation stimuli and primarily elicit responses over occipital scalp regions. A few studies have adopted spatial motion stimuli [20,21], which alter the pattern of visual responses and are associated with dorsal stream-related visual processing, but their performance remains limited. By contrast, text sequence stimulation evokes a broader scalp response distribution across occipital and occipitotemporal regions, which may provide greater design flexibility while maintaining high performance.

**Table 2.** Summary of subtle flicker brain–computer interface

Stimulus paradigm	Algorithm	Subject numbers	Target numbers	Accuracy /%	ITR /(bits·min^-1^)	Cue + Recognition time/s	Reference
High frequency	TRCA	12	40/72	96.77/86.23	119.05/95.68	0.5 + 3/3.5	[52]
High frequency	TDCA	16	40	88.87	51.83	1 + 4	[13]
High frequency	TRCA	21	12	84.62	153.79	0.5 + 0.6	[12]
High frequency	TRCA	17	4	87.75	16.73	1 + 4	[53]
High frequency	MFD	10	3	90.7	54.94	1.14	[54]
Low frequency	TRCA	10	12	94.90	64.35	1.5 + 1.5	[55]
Grid stimulus	TRCA	15	12/40	~	139/121	1 + 0.4/0.9	[14]
High duty cycle	~	6	6	82.08	25.08	3.66	[56]
Low contrast	TRCA	6	11	94.6	38.6	1 + 3	[57]
Low density	TRCA	12	8	90.10	73.12	0.9 + 1	[58]
SSMVEP	CCA	18	40	94	91.2	0.5 + 2.33	[20]
Gaiting stimulus	CCA	10	4	88.9	10.61	2 + 6	[21]
Spatial coding	ITCCA	13	13	90.3	43.8	0 + 4	[59]
Spatial coding	TRCA	17	4	87.50	22.48	0.5 + 3	[60]
**This study**	**TDCA**	**14**	**40**	**93.71**	**235.12**	**0.5 + 0.7**	**This study**

ITR, information transfer rate; SSMVEP, steady-state motion visual evoked potential; TRCA, task-related component analysis; TDCA, task-discriminant component analysis; MFD, match filter detector; CCA, canonical correlation analysis; ITCCA, individual-template canonical correlation analysis

Despite strong average performance, intersubject variability was observed, with online accuracies ranging from 79.06% to 100%. Importantly, this variability was not greater than that observed in conventional brightness paradigms (63.13% to 99.79% in our supplementary data). The variation may reflect individual differences in visual word processing, as seen in the amplitude and topography of responses (Fig. S10 to S12). Additionally, individual alpha rhythms influenced performance. For instance, participant Sub17 with strong alpha activity showed low classification accuracy, likely due to alpha-related interference [42]. Other factors such as reading fluency [43] and prior BCI experience [36] may also contribute. Addressing this variability may require both more advanced decoding algorithms and user-specific interface customization [44].

From the perspective of user experience, the perceptual characteristics of text sequence stimulation—small visual area, subtle luminance variation, and naturalistic transitions—may contribute to reduced flicker discomfort. Comfort was assessed using a 5-point questionnaire together with the NASA-TLX workload scale [13,19]. Results indicated a significant reduction in visual fatigue without a significant increase in subjective workload. Moreover, prolonged fixation under conventional brightness flicker stimulation may impose greater visual burden than under the proposed text sequence stimulation paradigm. However, these evaluations were obtained under controlled laboratory conditions and relatively short stimulation durations, which limits conclusions about cumulative fatigue in prolonged use.

In addition to supporting high-performance and comfortable BCI operation, the proposed text sequence stimulation paradigm also enabled us to investigate the response characteristics of text-evoked brain activity. In our study, frequency-dependent patterns were observed: Low-frequency stimuli (3 to 8 Hz) predominantly elicited stronger occipitotemporal scalp responses, whereas higher frequencies (>10 Hz) shifted responses toward more occipital-dominant patterns. Occipitotemporal responses peaked at 5.4 Hz and occipital responses at 11.8 Hz, suggesting that text stimuli recruit distinct response modes. Although EEG lacks the spatial resolution of functional magnetic resonance imaging or intracranial recordings, these patterns are consistent with prior neuroimaging evidence linking orthographic processing to visual processing related to the ventral stream [24,28,45]. Our source localization results (Fig. S5), although based on a standardized model, also suggested activity extending beyond early occipital regions into more extensive occipitotemporal areas. These observations raise the possibility that text sequence stimulation may differentially recruit processing stages beyond those typically emphasized by conventional brightness flicker paradigms, although this will require further testing with higher spatial resolution methods.

A related concern is whether the relatively small luminance differences between Chinese characters might reduce response SNR. While luminance contrast strongly influences conventional SSVEP paradigms [17], text sequence stimulation differs by combining periodic visual stimulation with orthographic-form information, resulting in distinct response characteristics. In our experiments manipulating stimulus size and luminance, we found that text sequence stimulation maintained relatively stable SNR across different conditions. At the same time, the present comparison should be interpreted at the paradigm level, where multiple stimulus properties jointly shape the response characteristics, rather than as evidence for a single isolated mechanism.

The present study still has several limitations that should be addressed in future work to better evaluate the practical performance of the proposed paradigm in real-world BCI applications. First, all participants were young, healthy, native Mandarin speakers, and the experiments were conducted in a controlled laboratory setting using a cue-guided synchronous spelling paradigm. Future studies should extend the evaluation to more realistic scenarios, including asynchronous free spelling, and to broader user populations such as patients and older adults.

In addition, the subjective comfort and NASA-TLX assessments in the present study were collected under relatively short stimulation durations. In practical use, especially for patient populations, the duration of use may be considerably longer. It will therefore be important to conduct more comprehensive long-term evaluations of factors such as fatigue accumulation, learning or adaptation effects, and age-related changes in reading ability, all of which may influence the usability and performance of the system. Reading proficiency and character familiarity were not directly quantified in this study, so their relationship with decoding performance could not be tested. However, prior work on orthographic processing suggests that familiarity with written forms may modulate neural responses to text stimuli [28]. This possibility is also consistent with part of our supplementary observations (Fig. S7 and S8). In the proposed paradigm, such an influence may be dynamic rather than fixed, because it could evolve with repeated exposure during use. Future studies will therefore be needed to quantify it more directly.

Beyond these experimental limitations, there is also room for further improvement in the performance and usability of text sequence stimulation paradigms. Possible directions include more efficient sequence design [4], semantic augmentation [46], and hybrid encoding methods [47] to alleviate harmonic overlap in JFPM-based systems. In addition, algorithmic improvements may be needed to better adapt to the dynamic spatial patterns of text-evoked responses, especially in the 10- to 12-Hz range, where transient and steady-state topographies differ [48]. Transfer learning and domain adaptation methods may also help reduce calibration requirements and thereby improve the practicality of the system [49].

More broadly, text sequence stimulation may offer unique advantages for wearable BCIs [50]. Conventional SSVEP paradigms using simple stimuli such as brightness modulation or patterned targets (e.g., checkerboards or Newton rings) often fail to elicit high SNR responses in hairless recording areas [14,20,36]. In contrast, text sequence stimulation can produce broader scalp response distributions, particularly in the 3- to 8-Hz range, extending beyond occipital scalp regions toward temporal and, in some cases, frontal scalp areas. Future work could therefore explore the integration of text sequence stimulation with electrodes placed in hairless areas, with the goal of developing more convenient and wearable BCI systems [50,51].

## Conclusion

In this study, we proposed a text sequence stimulation paradigm to achieve a better trade-off between BCI performance and visual comfort. Frequency-sweep analysis revealed clear frequency-dependent spatial patterns, with stronger occipitotemporal responses at lower frequencies and a gradual shift toward occipital dominance at higher frequencies. The proposed paradigm also showed greater robustness to stimulus size and luminance variations than conventional brightness flicker stimulation and supported a 40-target speller with an average online ITR of 235.12 bits/min ± 30.12 bits/min, together with improved subjective comfort. These findings suggest that text sequence stimulation can serve as a practical and comfortable alternative for high-speed visual BCI. Future work will focus on optimizing sequence design, reducing calibration requirements, and validating long-term usability in realistic and wearable settings.

## Data Availability

The data used to support the findings of this study are available from the corresponding authors upon request.

## Supplementary Materials

Supplementary 1 Figs. S1 to S12 Tables S1 and S2 Reference [61]

## References

[1] McFarland DJ, Wolpaw JR. EEG-based brain–computer interfaces. Curr Opin Biomed Eng. 2017;4:194–200. 29527584 10.1016/j.cobme.2017.11.004 PMC5839510

[2] Värbu K, Muhammad N, Muhammad Y. Past, present, and future of EEG-based BCI applications. Sensors. 2022;22(9):3331. 35591021 10.3390/s22093331 PMC9101004

[3] Chen X, Wang Y, Nakanishi M, Gao X, Jung T-P, Gao S. High-speed spelling with a noninvasive brain–computer interface. Proc Natl Acad Sci USA. 2015;112(44):E6058–E6067. 26483479 10.1073/pnas.1508080112 PMC4640776

[4] Shi N, Miao Y, Huang C, Li X, Song Y, Chen X, Wang Y, Gao X. Estimating and approaching the maximum information rate of noninvasive visual brain-computer interface. NeuroImage. 2024;289: Article 120548. 38382863 10.1016/j.neuroimage.2024.120548

[5] Zhang S, Chen Y, Zhang L, Gao X, Chen X. Study on robot grasping system of SSVEP-BCI based on augmented reality stimulus. Tsinghua Sci Technol. 2023;28(2):322–329.

[6] Adams M, Benda M, Saboor A, Krause AF, Rezeika A, Gembler F, Stawicki P, Hesse M, Essig K, Ben-Salem S, et al. Towards an SSVEP-BCI controlled smart home. In: *2019 IEEE International Conference on Systems, Man and Cybernetics (SMC)*. IEEE; 2019. p. 2737–2742.

[7] Chen Y, Yang C, Ye X, Chen X, Wang Y, Gao X. Implementing a calibration-free SSVEP-based BCI system with 160 targets. J Neural Eng. 2021;18(4): Article 046094. 10.1088/1741-2552/ac0bfa 34134091

[8] Zhang S, Gao X, Chen X. Humanoid robot walking in maze controlled by SSVEP-BCI based on augmented reality stimulus. Front Hum Neurosci. 2022;16: Article 908050. 35911600 10.3389/fnhum.2022.908050 PMC9330178

[9] Shi N, Wang L, Chen Y, Yan X, Yang C, Wang Y, Gao X. Steady-state visual evoked potential (SSVEP)-based brain–computer interface (BCI) of Chinese speller for a patient with amyotrophic lateral sclerosis: A case report. J Neuro-Oncol. 2020;8(1):40–52.

[10] Zhu D, Bieger J, Garcia Molina G, Aarts RM. A survey of stimulation methods used in SSVEP-based BCIs. Comput Intell Neurosci. 2010;2010(1): Article 702357. 20224799 10.1155/2010/702357 PMC2833411

[11] Xie J, Xu G, Wang J, Li M, Han C, Jia Y. Effects of mental load and fatigue on steady-state evoked potential based brain computer Interface tasks: A comparison of periodic flickering and motion-reversal based visual attention. PLOS One. 2016;11(9): Article e0163426. 27658216 10.1371/journal.pone.0163426 PMC5033480

[12] Chen X, Liu B, Wang Y, Cui H, Dong J, Ma R, Li N, Gao X. Optimizing stimulus frequency ranges for building a high-rate high frequency SSVEP-BCI. IEEE Trans Neural Syst Rehabil Eng. 2023;31:1277–1286. 37022899 10.1109/TNSRE.2023.3243786

[13] Liu K, Yao Z, Zheng L, Wei Q, Pei W, Gao X, Wang Y. A high-frequency SSVEP-BCI system based on a 360 Hz refresh rate. J Neural Eng. 2023;20(4): Article 046042. 10.1088/1741-2552/acf242 37604119

[14] Ming G, Zhong H, Pei W, Gao X, Wang Y. A new grid stimulus with subtle flicker perception for user-friendly SSVEP-based BCIs. J Neural Eng. 2023;20(2): Article 026010. 10.1088/1741-2552/acbee0 36827704

[15] Ming G, Pei W, Chen H, Gao X, Wang Y. Optimizing spatial properties of a new checkerboard-like visual stimulus for user-friendly SSVEP-based BCIs. J Neural Eng. 2021;18(5): Article 056046. 10.1088/1741-2552/ac284a 34544060

[16] Gilford C, Camilleri T, Camilleri KP. User discomfort in SSVEP-based BCIs - can modulation depth offer a solution? Brain-Comput Interfaces. 2024;11(4):159–177.

[17] Si-Mohammed H, Holz C, Wilson A, Gamper H, Lee AK, Emmanouilidou D, Cutrell E, Tashev I. On the effect of size and contrast of the SSVEP visual stimuations on classification accuracy and user-friendliness in virtual reality. In: *2023 11th International Winter Conference on Brain-Computer Interface (BCI)*. IEEE; 2023. p. 1–6.

[18] Floriano A, Diez PF, Freire Bastos-Filho T. Evaluating the influence of chromatic and luminance stimuli on SSVEPs from behind-the-ears and occipital areas. Sensors. 2018;18(2):615. 29462975 10.3390/s18020615 PMC5855130

[19] Zheng X, Xu G, Zhang Y, Liang R, Zhang K, Du Y, Xie J, Zhang S. Anti-fatigue performance in SSVEP-based visual acuity assessment: A comparison of six stimulus paradigms. Front Hum Neurosci. 2020;14:301. 32848675 10.3389/fnhum.2020.00301 PMC7412756

[20] Han C, Xu G, Xie J, Chen C, Zhang S. Highly interactive brain–computer interface based on flicker-free steady-state motion visual evoked potential. Sci Rep. 2018;8(1):5835. 29643430 10.1038/s41598-018-24008-8 PMC5895715

[21] Zhang X, Xu G, Ravi A, Pearce S, Jiang N. Can a highly accurate multi-class SSMVEP BCI induce sensory-motor rhythm in the sensorimotor area? J Neural Eng. 2021;18(3): Article 035001. 10.1088/1741-2552/ab85b2 32238617

[22] Song Y, Liu B, Li X, Shi N, Wang Y, Gao X. Decoding natural images from EEG for object recognition. arXiv. 2024. 10.48550/arXiv.2308.13234

[23] Leong D, Do TT-T, Lin C-T. Ventral and dorsal stream EEG channels: Key features for EEG-based object recognition and identification. IEEE Trans Neural Syst Rehabil Eng. 2023;31:4862–4870. 38051624 10.1109/TNSRE.2023.3339698

[24] Brem S, Halder P, Bucher K, Summers P, Martin E, Brandeis D. Tuning of the visual word processing system: Distinct developmental ERP and fMRI effects. Hum Brain Mapp. 2009;30(6):1833–1844. 19288464 10.1002/hbm.20751 PMC6871060

[25] Freiwald WA, Tsao DY. Functional compartmentalization and viewpoint generalization within the macaque face-processing system. Science. 2010;330(6005):845–851. 21051642 10.1126/science.1194908 PMC3181095

[26] Rolls ET. Invariant visual object and face recognition: Neural and computational bases, and a model, VisNet. Front Comput Neurosci. 2012;6:35. 22723777 10.3389/fncom.2012.00035 PMC3378046

[27] Dehaene S, Cohen L. The unique role of the visual word form area in reading. Trends Cogn Sci. 2011;15(6):254–262. 21592844 10.1016/j.tics.2011.04.003

[28] Woolnough O, Donos C, Rollo PS, Forseth KJ, Lakretz Y, Crone NE, Fischer-Baum S, Dehaene S, Tandon N. Spatiotemporal dynamics of orthographic and lexical processing in the ventral visual pathway. Nat Hum Behav. 2020;5(3):389–398. 33257877 10.1038/s41562-020-00982-w PMC10365894

[29] Bi Y, Wang X, Caramazza A. Object domain and modality in the ventral visual pathway. Trends Cogn Sci. 2016;20(4):282–290. 26944219 10.1016/j.tics.2016.02.002

[30] Zhang Y, Zhao Q, Jing J, Wang X, Cichocki A. A novel BCI based on ERP components sensitive to configural processing of human faces. J Neural Eng. 2012;9(2): Article 026018. 22414683 10.1088/1741-2560/9/2/026018

[31] Jin J, Allison BZ, Kaufmann T, Kübler A, Zhang Y, Wang X, Cichocki A. The changing face of P300 BCIs: A comparison of stimulus changes in a P300 BCI involving faces, emotion, and movement. PLOS One. 2012;7(11): Article e49688. 23189154 10.1371/journal.pone.0049688 PMC3506655

[32] Lochy A, Van Belle G, Rossion B. A robust index of lexical representation in the left occipito-temporal cortex as evidenced by EEG responses to fast periodic visual stimulation. Neuropsychologia. 2015;66:18–31. 25448857 10.1016/j.neuropsychologia.2014.11.007

[33] Lochy A, Van Reybroeck M, Rossion B. Left cortical specialization for visual letter strings predicts rudimentary knowledge of letter-sound association in preschoolers. Proc Natl Acad Sci USA. 2016;113(30):8544–8549. 27402739 10.1073/pnas.1520366113 PMC4968710

[34] Mercure E, Kadosh KC, Johnson MH. The N170 shows differential repetition effects for faces, objects, and orthographic stimuli. Front Hum Neurosci. 2011;5:6. 21283529 10.3389/fnhum.2011.00006 PMC3031024

[35] Guger C, Edlinger G, Harkam W, Niedermayer I, Pfurtscheller G. How many people are able to operate an EEG-based brain-computer interface (BCI)? IEEE Trans Neural Syst Rehabil Eng. 2003;11(2):145–147. 12899258 10.1109/TNSRE.2003.814481

[36] Wang Y, Chen X, Gao X, Gao S. A Benchmark Dataset for SSVEP-Based Brain–Computer Interfaces. IEEE Trans Neural Syst Rehabil Eng. 2017;25(10):1746–1752. 27849543 10.1109/TNSRE.2016.2627556

[37] Chen X, Wang Y, Gao S, Jung T-P, Gao X. Filter bank canonical correlation analysis for implementing a high-speed SSVEP-based brain–computer interface. J Neural Eng. 2015;12(4): Article 046008. 26035476 10.1088/1741-2560/12/4/046008

[38] Liu B, Chen X, Shi N, Wang Y, Gao S, Gao X. Improving the performance of individually calibrated SSVEP-BCI by task-discriminant component analysis. IEEE Trans Neural Syst Rehabil Eng. 2021;29:1998–2007. 34543200 10.1109/TNSRE.2021.3114340

[39] Nakanishi M, Wang Y, Chen X, Wang Y-T, Gao X, Jung T-P. Enhancing detection of SSVEPs for a high-speed brain speller using task-related component analysis. IEEE Trans Biomed Eng. 2018;65(1):104–112. 28436836 10.1109/TBME.2017.2694818 PMC5783827

[40] Mazade R, Jin J, Rahimi-Nasrabadi H, Najafian S, Pons C, Alonso J-M. Cortical mechanisms of visual brightness. Cell Rep. 2022;40(13): Article 111438. 36170812 10.1016/j.celrep.2022.111438 PMC9552773

[41] Han Y, Roig G, Geiger G, Poggio T. Scale and translation-invariance for novel objects in human vision. Sci Rep. 2020;10(1):1411. 31996698 10.1038/s41598-019-57261-6 PMC6989457

[42] Vialatte F-B, Maurice M, Dauwels J, Cichocki A. Steady-state visually evoked potentials: Focus on essential paradigms and future perspectives. Prog Neurobiol. 2010;90(4):418–438. 19963032 10.1016/j.pneurobio.2009.11.005

[43] Dehaene-Lambertz G, Monzalvo K, Dehaene S. The emergence of the visual word form: Longitudinal evolution of category-specific ventral visual areas during reading acquisition. PLoS Biol. 2018;16(3): Article e2004103. 29509766 10.1371/journal.pbio.2004103 PMC5856411

[44] Liang L, Bin G, Chen X, Wang Y, Gao S, Gao X. Optimizing a left and right visual field biphasic stimulation paradigm for SSVEP-based BCIs with hairless region behind the ear. J Neural Eng. 2021;18(6): Article 066040. 10.1088/1741-2552/ac40a1 34875637

[45] Pleisch G, Karipidis II, Brem A, Röthlisberger M, Roth A, Brandeis D, Walitza S, Brem S. Simultaneous EEG and fMRI reveals stronger sensitivity to orthographic strings in the left occipito-temporal cortex of typical versus poor beginning readers. Dev Cogn Neurosci. 2019;40: Article 100717. 31704655 10.1016/j.dcn.2019.100717 PMC6974919

[46] Ding N, Melloni L, Yang A, Wang Y, Zhang W, Poeppel D. Characterizing neural entrainment to hierarchical linguistic units using electroencephalography (EEG). Front Hum Neurosci. 2017;11:481. 29033809 10.3389/fnhum.2017.00481 PMC5624994

[47] Han J, Xu M, Xiao X, Yi W, Jung T-P, Ming D. A high-speed hybrid brain-computer interface with more than 200 targets. J Neural Eng. 2023;20(1): Article 016025. 10.1088/1741-2552/acb105 36608342

[48] Liu X, Liu B, Dong G, Gao X, Wang Y. Facilitating applications of SSVEP-based BCIs by within-subject information transfer. Front Neurosci. 2022;16: Article 863359. 35720721 10.3389/fnins.2022.863359 PMC9198902

[49] Miao Y, Shi N, Huang C, Song Y, Chen X, Wang Y, Gao X. High-performance c-VEP-BCI under minimal calibration. Expert Syst Appl. 2024;249: Article 123679.

[50] Wang Z, Shi N, Zhang Y, Zheng N, Li H, Jiao Y, Cheng J, Wang Y, Zhang X, Chen Y, et al. Conformal in-ear bioelectronics for visual and auditory brain-computer interfaces. Nat Commun. 2023;14(1):4213. 37452047 10.1038/s41467-023-39814-6 PMC10349124

[51] Ali A, Ullah I, Kumar Singh S, Jiang W, Alturise F, Bai X. Attention-driven graph convolutional networks for deadline-constrained virtual machine task allocation in edge computing. IEEE Trans Consum Electron. 2025;71(2):5595–5605.

[52] Ye X, Yang C, Chen Y, Wang Y, Gao X, Zhang H. Multisymbol time division coding for high-frequency steady-state visual evoked potential-based brain-computer Interface. IEEE Trans Neural Syst Rehabil Eng. 2022;30:1693–1704. 35714087 10.1109/TNSRE.2022.3183087

[53] Jiang L, Pei W, Wang Y. A user-friendly SSVEP-based BCI using imperceptible phase-coded flickers at 60Hz. China Commun. 2022;19(2):1–14.

[54] Hsu C-C, Yeh C-L, Lee W-K, Hsu H-T, Shyu K-K, Li LP-H, Wu T-Y, Lee P-L. Extraction of high-frequency SSVEP for BCI control using iterative filtering based empirical mode decomposition. Biomed Signal Process Control. 2020;61: Article 102022.

[55] Jiang L, Li X, Pei W, Gao X, Wang Y. A hybrid brain-computer interface based on visual evoked potential and pupillary response. Front Hum Neurosci. 2022;16: Article 834959. 35185500 10.3389/fnhum.2022.834959 PMC8850273

[56] Lee P-L, Yeh C-L, Cheng JY-S, Yang C-Y, Lan G-Y. An SSVEP-based BCI using high duty-cycle visual flicker. IEEE Trans Biomed Eng. 2011;58(12):3350–3359. 21788179 10.1109/TBME.2011.2162586

[57] Ladouce S, Darmet L, Torre Tresols JJ, Velut S, Ferraro G, Dehais F. Improving user experience of SSVEP BCI through low amplitude depth and high frequency stimuli design. Sci Rep. 2022;12(1):8865. 35614168 10.1038/s41598-022-12733-0 PMC9132909

[58] Meng J, Liu H, Wu Q, Zhou H, Shi W, Meng L, Xu M, Ming D. A SSVEP-based brain–computer interface with low-pixel density of stimuli. IEEE Trans Neural Syst Rehabil Eng. 2023;31:4439–4448. 37906489 10.1109/TNSRE.2023.3328917

[59] Chen J, Wang Y, Maye A, Hong B, Gao X, Engel AK, Zhang D. A spatially-coded visual brain-computer Interface for flexible visual spatial information decoding. IEEE Trans Neural Syst Rehabil Eng. 2021;29:926–933. 33983885 10.1109/TNSRE.2021.3080045

[60] Hu R, Ming G, Wang Y, Gao X. A sub-region combination scheme for spatial coding in a high-frequency SSVEP-based BCI. J Neural Eng. 2023;20(4): Article 046018. 10.1088/1741-2552/ace8bd 37467742

[61] Tadel F, Baillet S, Mosher JC, Pantazis D, Leahy RM. Brainstorm: A user-friendly application for MEG/EEG analysis. Comput Intell Neurosci. 2011;2011:879716. 21584256 10.1155/2011/879716 PMC3090754

