# Supplementary material for: Text Sequence Stimulation for High-Speed and Comfortable SSVEP-BCI
Source: Cyborg Bionic Syst. 2026 Jun 15;7:0612. doi: 10.34133/cbsystems.0612 (PMC13266052; doi:10.34133/cbsystems.0612)
Supplement: Supplementary 1 — Figs. S1 to S12 Tables S1 and S2 Reference [61] [file cbsystems.0612.f1.docx]

SUPPLEMENTARY MATERIALS

**Experimental procedure**

*
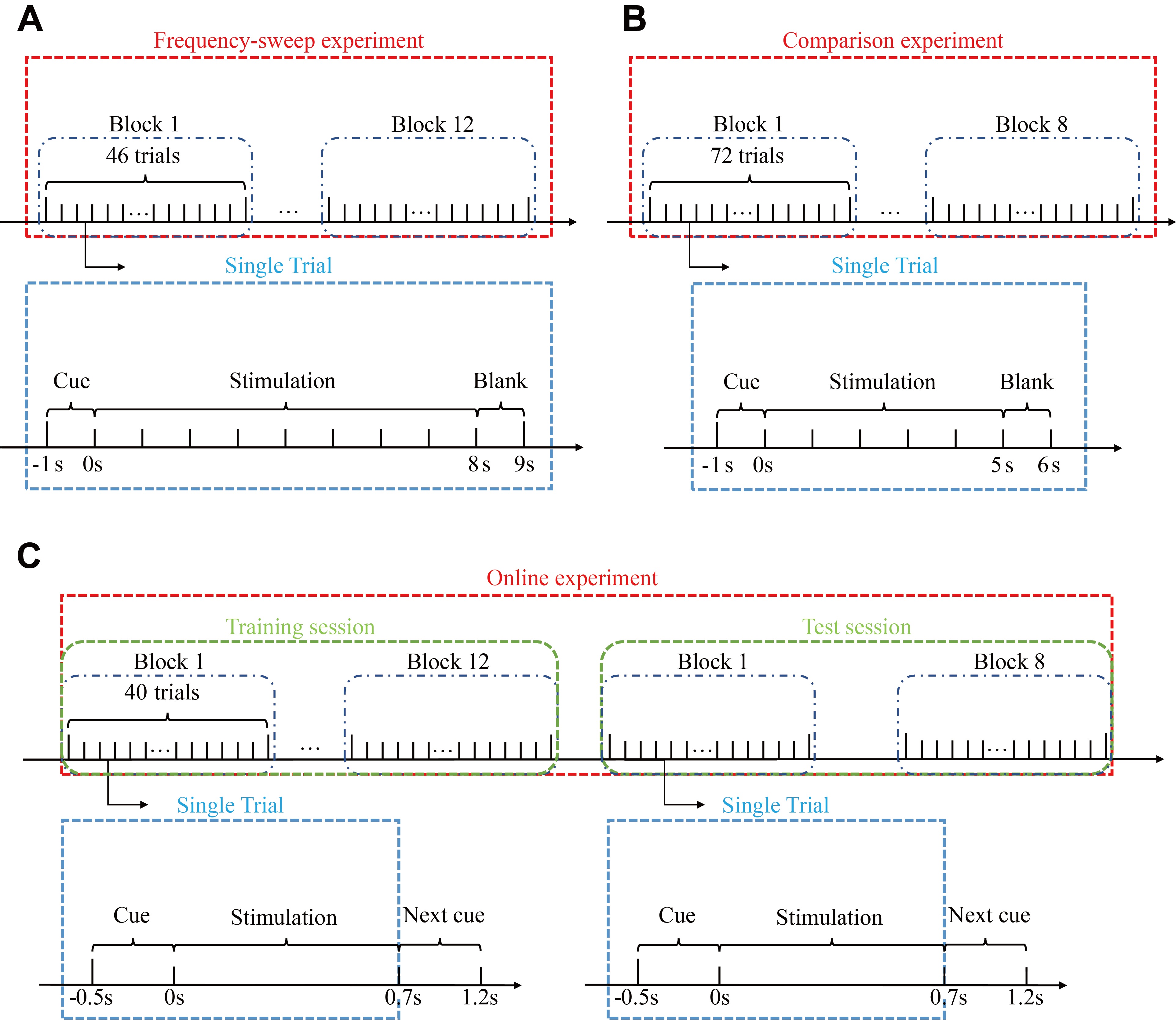
*

***Fig S1.*** *Experimental procedures of the (A) frequency-sweep experiment(B) comparison experiment and (C) online experiments.*

**System resources**

Both the offline analysis and online decoding procedures were implemented in MATLAB (MathWorks, USA) on a desktop workstation (Dell Precision 5820 Tower) equipped with an Intel(R) Xeon(R) W-2135 @ 3.70 GHz , NVIDIA GeForce RTX 2080 Ti and 32 GB RAM. No GPU acceleration was used.

**Recognition procedure**

*
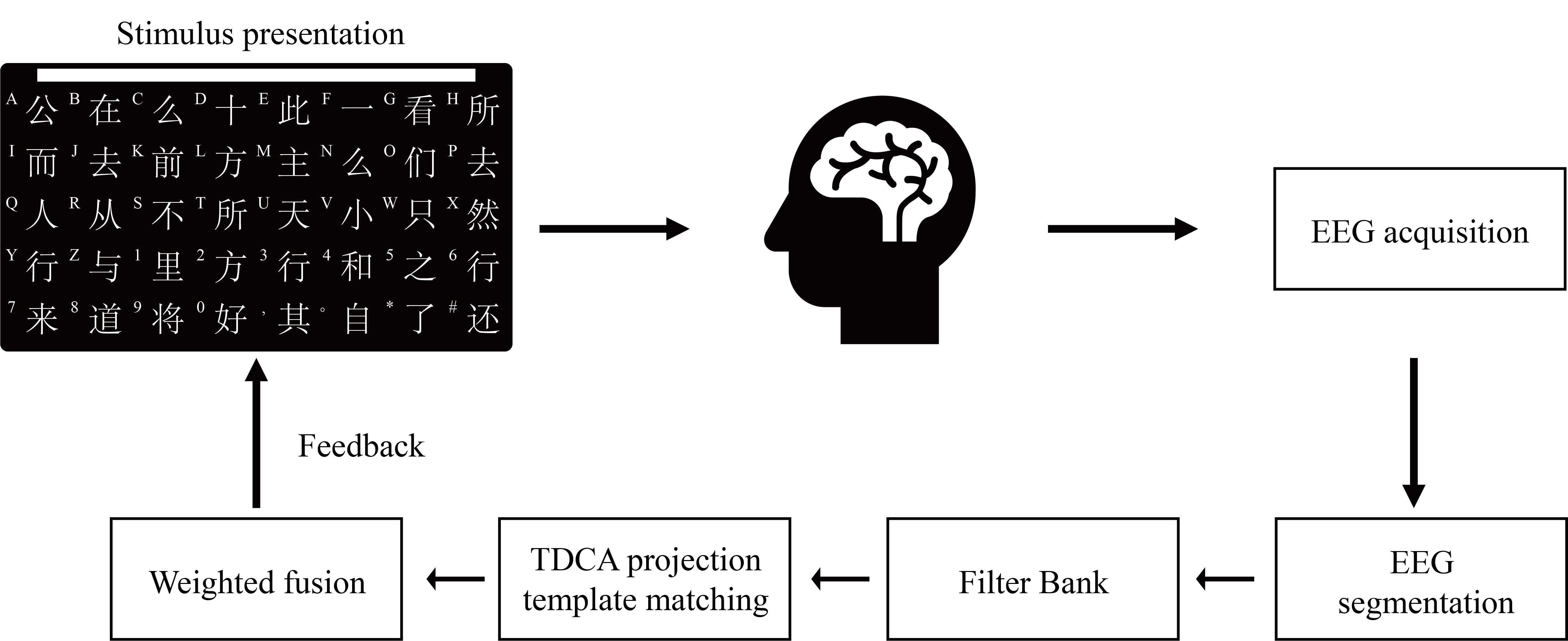
*

***Fig S2****. Overall workflow of the BCI system.*

During the decoding process of the offline frequency-sweep experiment, we performed phase simulation on the offline data to ensure that the phase intervals were consistent with those used in the online experiment, as illustrated in Fig. S3. Since the original data corresponded to the zero-phase condition and SSVEP responses are phase locked to the stimulus, shifting the signal in time by $\tau_{i}$allows us to obtain the EEG response corresponding to a target phase. The value of $\tau_{i}$ is defined by Equation (13).

**
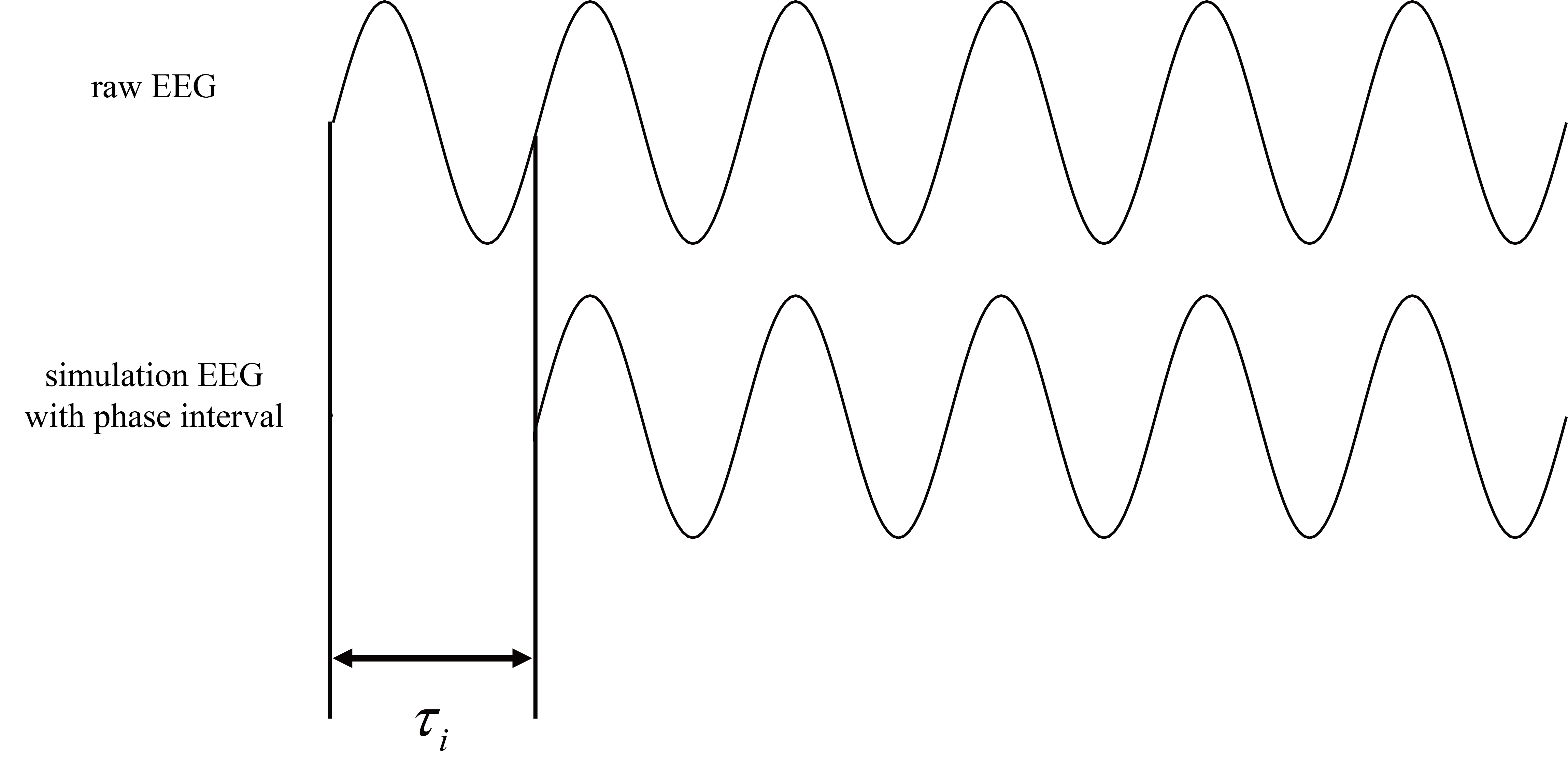
**

***Fig S3.*** *Schematic illustration of phase-interval simulation in EEG data.*

***Table S1.*** *Filter Bank setting*

| Sub-band | Lower cutoff frequency (Hz) | Upper cutoff frequency  (Hz) |
| --- | --- | --- |
| 1 | 3.4-1 | 26 |
| 2 | 2*3.4-1 | 26 |
| 3 | 3*3.4-1 | 26 |
| 4 | 4*3.4-1 | 26 |
| 5 | 5*3.4-1 | 26 |

**Parameter optimization**

Fig. S4 illustrates the optimization of classification parameters for the 40-target system, including the number of sub-bands, the number of electrodes, the number of training data, and sub-bands weighting coefficients. The number of sub-bands, electrodes, and training data significantly affect classification performance. This study compared the performance differences between the TRCA and TDCA algorithms under various classification parameters. TDCA consistently outperformed TRCA across all parameters. Classification accuracy are evaluated using leave-one-out cross-validation, with training data consisting of 11 trials.

Fig. S4A shows the impact of different numbers of electrodes on the decoding performance of the TDCA and TRCA algorithms. The classification performance of the TRCA algorithm significantly improves with an increasing number of electrodes (paired t-test, p<0.05). For the TDCA algorithm, performance increases with the number of electrodes up to 39 channels, after which the performance no longer shows a significant increase (paired t-test, p>0.05). Fig. S4B illustrates the impact of different number of sub-bands on the decoding performance of the TDCA and TRCA algorithms. The classification performance of the TRCA algorithm significantly improves with an increasing number of sub-bands (paired t-test, p<0.05). The TDCA algorithm's performance significantly improves when the number of sub-bands increases from 1 to 5 (paired t-test, p<0.05), but slightly decreases when the number of sub-bands continues to increase (p>0.05). Fig. S4C demonstrates the effect of different numbers of training data on the decoding performance of the TDCA and TRCA algorithms. As the training data volume increases, the classification accuracy of both TRCA and TDCA algorithms increases. Different sub-bands were weighted according$w=n^{-a}+b$ (n is the number of sub-bands). Fig. S4D shows the classification accuracy under different parameters of a and b. The highest classification accuracy is achieved under the condition of a=2, b=0.1.


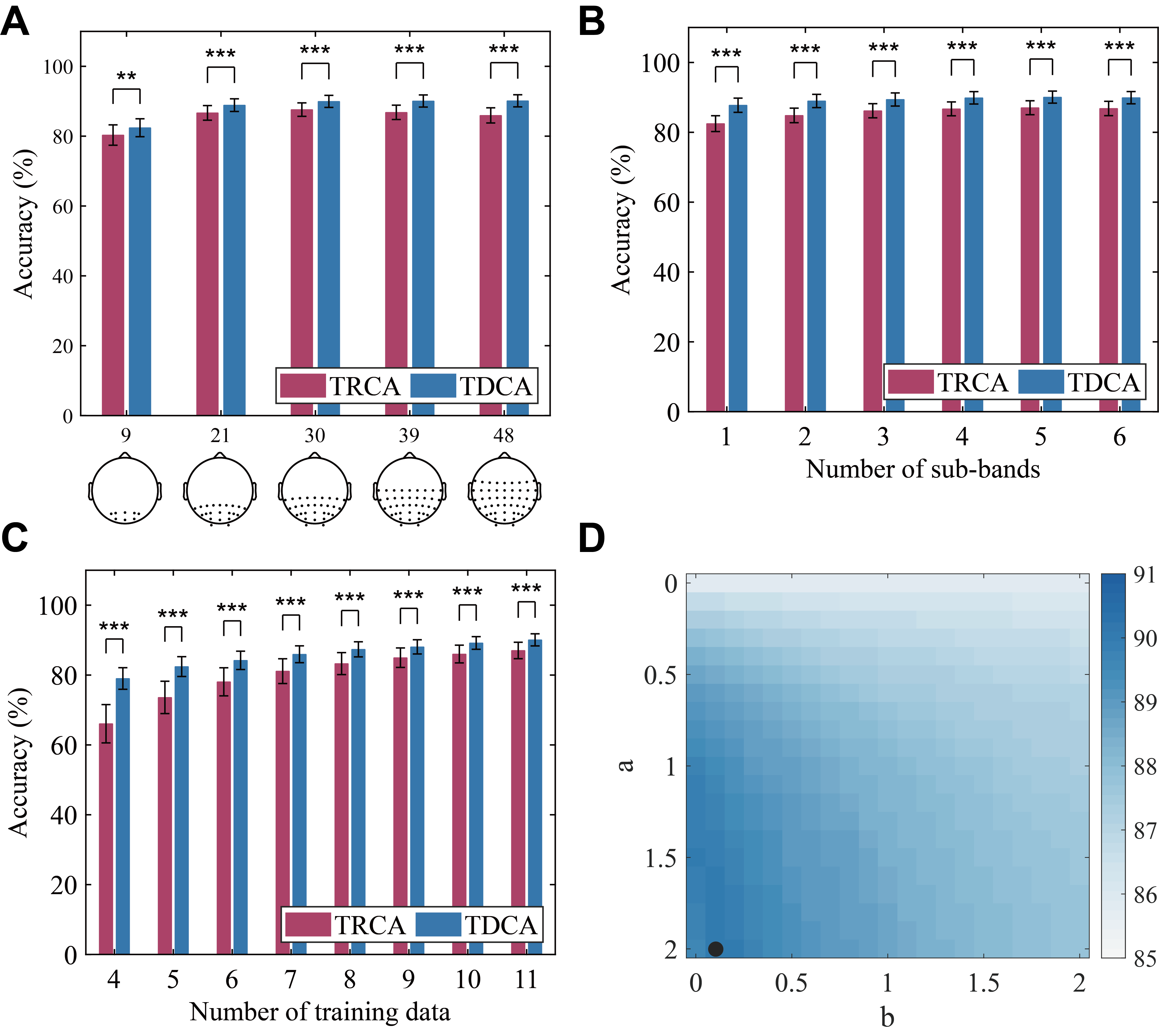


***Fig. S4.*** *Classification accuracy of TRCA and TDCA under different parameters. (A) Number of electrodes, (B) Number of sub-bands, (C) Number of training data (paired t-test, * p<0.05, ** p<0.01, *** p<0.001), (D) Sub-band weighting coefficients, with the black circle representing the optimal parameters. The colorbar indicates the classification accuracy under different weights.*

**Source localization of text evoked response**

Source-level analysis was conducted using the standardized low-resolution brain electromagnetic tomography (sLORETA) algorithm implemented in Brainstorm[61]. Scalp EEG was projected onto the default MNI/ICBM152 template brain, and source-level SNR maps were computed under the 6 Hz and 12 Hz text stimulation conditions. Because no individual MRI or digitized sensor positions were available, this analysis was intended as an exploratory source-level visualization rather than a basis for anatomical localization.

As shown in Supplementary Fig. S5A and S5B, the source-level SNR maps under 6 Hz and 12 Hz text stimulation exhibited different spatial patterns. In this exploratory analysis, the 6 Hz condition appeared more spatially distributed, whereas the 12 Hz condition appeared relatively more concentrated in posterior visual areas. Supplementary Fig. S5C and S5D further illustrate the source-level SNR profiles for right inferior temporal regions defined on the template source space. These observations are presented only as source-level visualizations and should not be interpreted as precise anatomical localization.

Although this exploratory source-level analysis was performed on a standard brain template without individual anatomical data, the overall spatial patterns were broadly consistent with the distributed scalp-level observations reported in the main text. However, these results are not sufficient for anatomical inference or for claims regarding pathway-specific processing.**
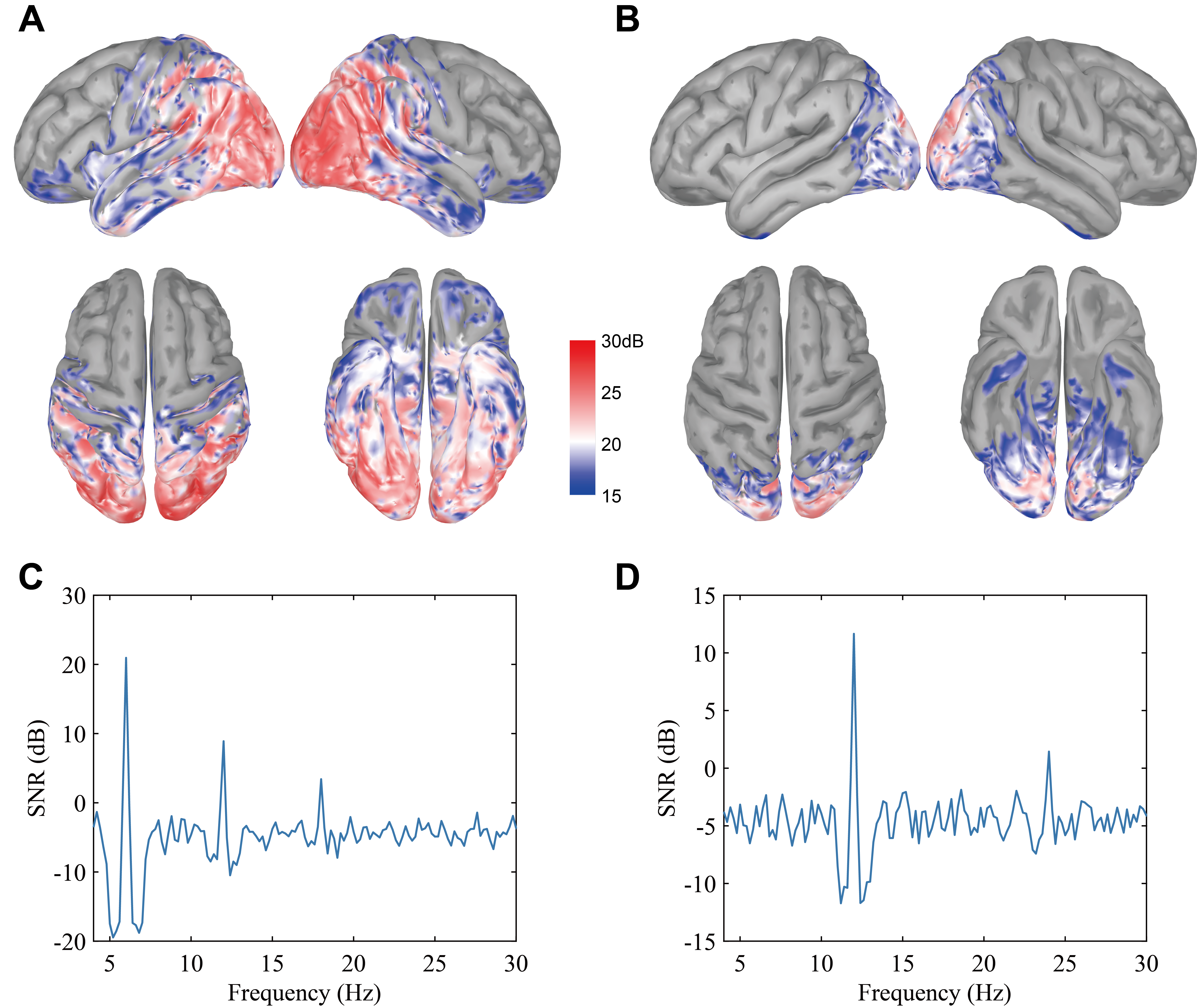
**

***Fig. S5.*** *Exploratory source-level visualization using sLORETA. (A-B) Source-level SNR maps for 6 Hz and 12 Hz stimulation. (C-D) Source-level SNR profiles for right inferior temporal regions defined on the template source space for 6 Hz and 12 Hz stimulation. These results are provided for illustrative purposes only and are not intended for anatomical localization.*

**Experiment on character manipulation**

To better understand how different character properties and levels of structural disruption affect neural responses, we conducted an additional character manipulation experiment. The experiment included three categories of Chinese characters and three corresponding structure-manipulated conditions. The three character categories were: (1) common characters with a moderate stroke count, selected from the Level-1 Chinese character database, with a mean stroke count of 6.06; (2) common characters with a high stroke count, also selected from the Level-1 database, with a mean stroke count of 10.60; and (3) uncommon characters with a moderate stroke count, selected from the Level-3 database, with a mean stroke count of 6.10.

Based on the common characters with a moderate stroke count, three additional structure-manipulated conditions were generated to progressively disrupt global character structure while preserving local pixel information. The first condition was inverted characters, which preserved the original character composition but altered its spatial arrangement. The second condition was a 2 × 2 scrambling, in which each character image was divided into four equal blocks that were randomly rearranged and reassembled. The third condition was a 10 × 10 scrambling, in which each character image was divided into 100 equal blocks that were randomly rearranged and reassembled. During scrambling, the pixel values within each block were kept unchanged, so that overall luminance and contrast were approximately preserved while the global character configuration was progressively disrupted.

The experiment consisted of 8 blocks, each containing all stimulus conditions. For each condition, responses were tested at four stimulation frequencies (3, 6, 9, and 12 Hz). Each block therefore contained 24 trials (6 conditions × 4 frequencies), and the order of stimulus condition and frequency was randomized within each block. For a given condition and stimulation frequency, the presentation order of specific exemplars was kept identical across repetitions. Each trial began with a 0.5 s fixation cross, followed by 5 s of visual stimulation, and ended with a 0.5 s blank interval, as shown in Fig. S6.


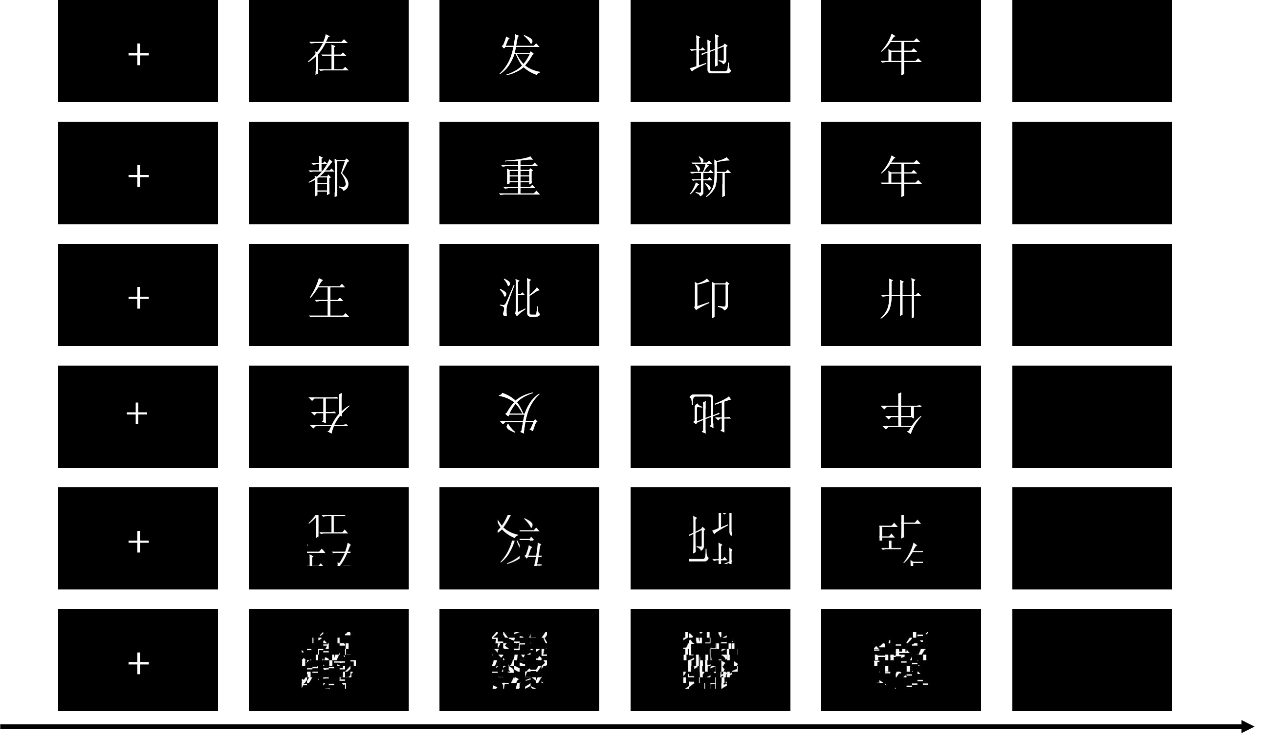


***Fig. S6.*** *Schematic illustration of a single trial under different stimulus conditions.*

A total of 12 participants took part in the experiment (5 females; age range: 21–32 years; mean age: 25.58 years). All participants were native Chinese speakers with normal or corrected-to-normal vision. The data acquisition and processing procedures were identical to those described in the main manuscript.

As shown in Fig. S7, all six character conditions exhibited a prominent SNR peak around 6 Hz, suggesting that this frequency range was the most effective for eliciting steady-state responses in the present experiment. Intact common characters produced the highest SNRs overall, while the effect of stroke count on SNR was relatively small. Compared with the common (moderate) condition, the uncommon (moderate) condition showed a significantly higher SNR at 3 Hz, but significantly lower SNRs at 6 Hz and 9 Hz. At 12 Hz, the difference between the two conditions was reduced to 0.37 dB and was no longer significant.

When the overall character structure was preserved but the characters were inverted, the SNRs were consistently lower than those of the upright condition, with significant decreases at 6 Hz and 9 Hz. The two scrambled-character conditions also showed consistently reduced responses relative to intact characters. Specifically, the 2 × 2 scrambled condition showed a significant decrease at 6 Hz, whereas the 10 × 10 scrambled condition showed significant decreases at 6 Hz and 9 Hz. However, no significant difference was observed between the 2 × 2 scrambled and 10 × 10 scrambled conditions.

Overall, these results suggest that preserving global character structure is associated with stronger steady-state responses, particularly near the optimal response frequency. This effect may also be influenced by factors such as character familiarity and presentation orientation. At the same time, the magnitude of the effect varied across stimulation frequencies, suggesting that the influence of character properties and levels of structural disruption on SSVEP responses is frequency dependent.


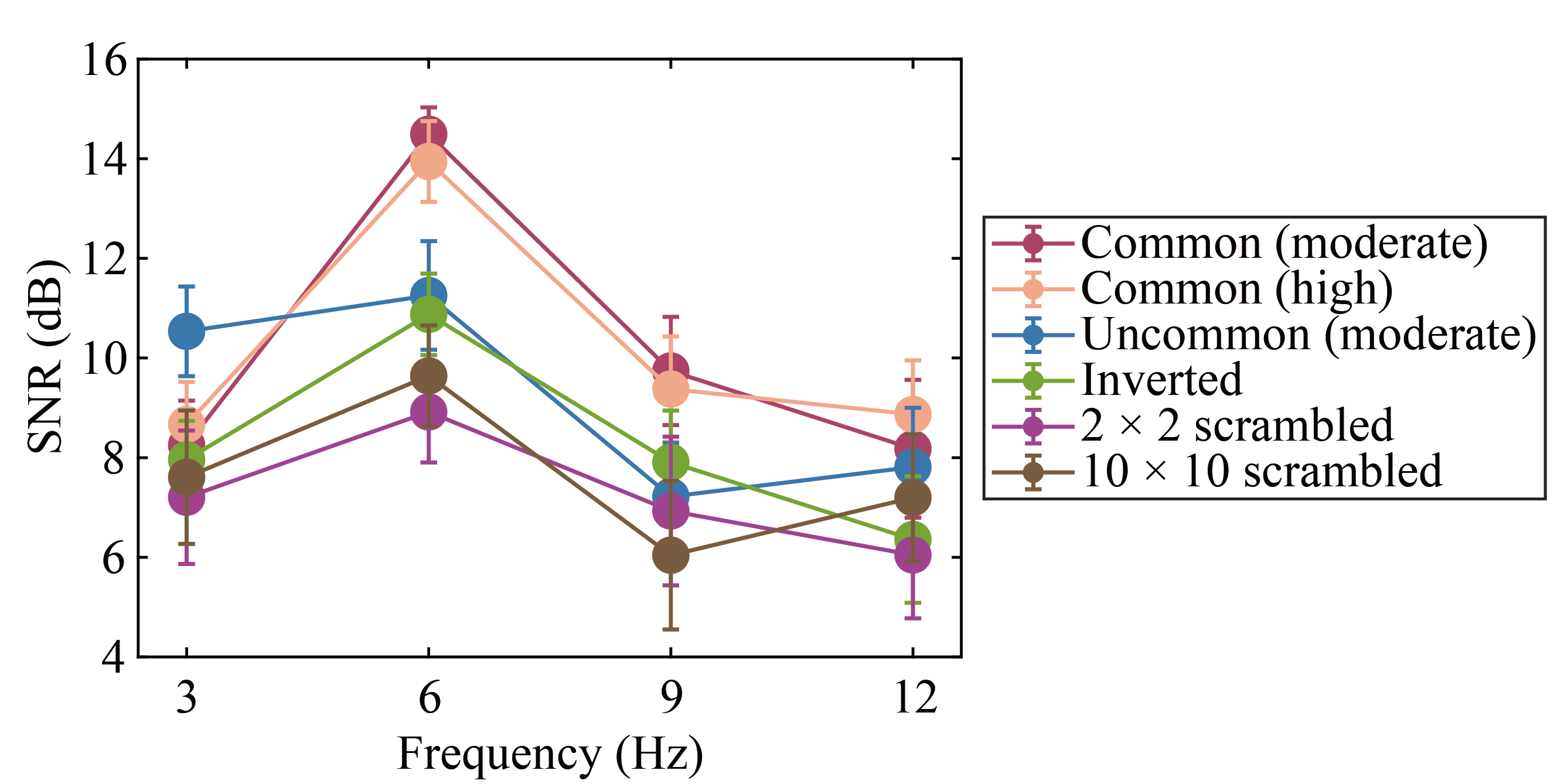


***Fig. S7.*** *SNR under different conditions across stimulation frequencies (3, 6, 9, and 12 Hz).* *Error bars indicate standard error across subjects.*

The scalp topographies in Fig. S8 showed corresponding differences in spatial distribution across character conditions. Intact character conditions generally elicited clearer posterior response patterns, especially at 6 Hz, whereas the effect of stroke count on the overall topographic pattern appeared relatively small. The uncommon character condition showed response patterns at 3 Hz and 6 Hz that were broadly similar to the 6 Hz pattern observed in the common character conditions. Under the inverted condition, the overall topographic pattern remained broadly similar to that of the upright condition, while the corresponding SNR values were reduced. In contrast, the scrambled character conditions showed weaker and less differentiated topographies overall.

Together, these results suggest that character properties and levels of structural disruption influence both the strength and spatial organization of the steady-state response. In particular, the effect of stroke count appears to be relatively limited, whereas extremely unfamiliar characters may affect response strength within certain frequency ranges. However, because the present manipulation does not fully isolate all lower-level visual properties, these findings should be interpreted as partial support for a contribution from structured character form, rather than as definitive causal evidence. Further work is needed to clarify which specific properties of text stimuli are most beneficial for neural encoding in visual BCI paradigms.

*
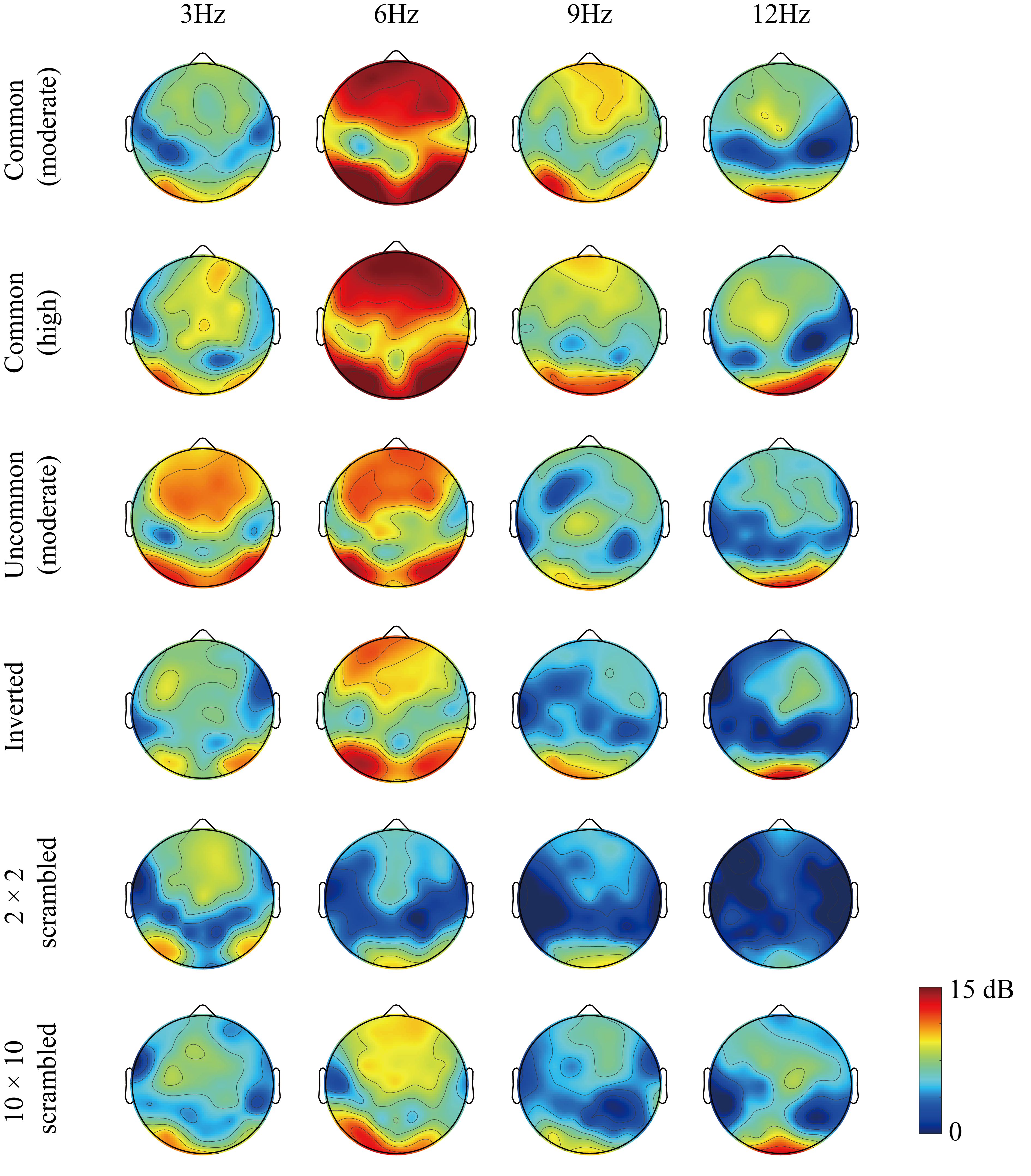
*

*Fig. S8. Scalp topographies for different character conditions at 3, 6, 9, and 12 Hz.*

**Performance comparison of two paradigms**

To provide a comprehensive comparison, we conducted an additional experiment using a conventional 40-target SSVEP speller. Stimuli were presented at frequencies ranging from 8 to 15.8 Hz[39], with each trial consisting of a 0.5 s cue followed by 0.5 s stimulation, across 12 blocks of data. The decoding parameters were consistent with those described in previous studies[38]. For performance evaluation, a leave-one-block-out cross-validation procedure was applied, with 11 blocks used for training and the remaining block for testing.

The results showed that although the text sequence paradigm achieved a competitive ITR, there remained a performance gap of approximately 20 bits/min compared with the conventional SSVEP paradigm (Fig. S9A). Specifically, under 0.5 s stimulation, no statistically significant difference in classification accuracy was observed between the two paradigms, indicating comparable decoding precision (Fig. S9B). Both paradigms exhibited noticeable inter-subject variability. Classification accuracy for conventional SSVEP stimuli ranged from 63.13% to 99.79%, while for text sequence stimuli, it ranged from 77.50% to 99.79%.

These findings suggest that while conventional SSVEP paradigms retain a performance advantage, text-based stimulation achieves comparable decoding accuracy with higher comfort, thereby offering a more balanced trade-off between performance and usability.

***
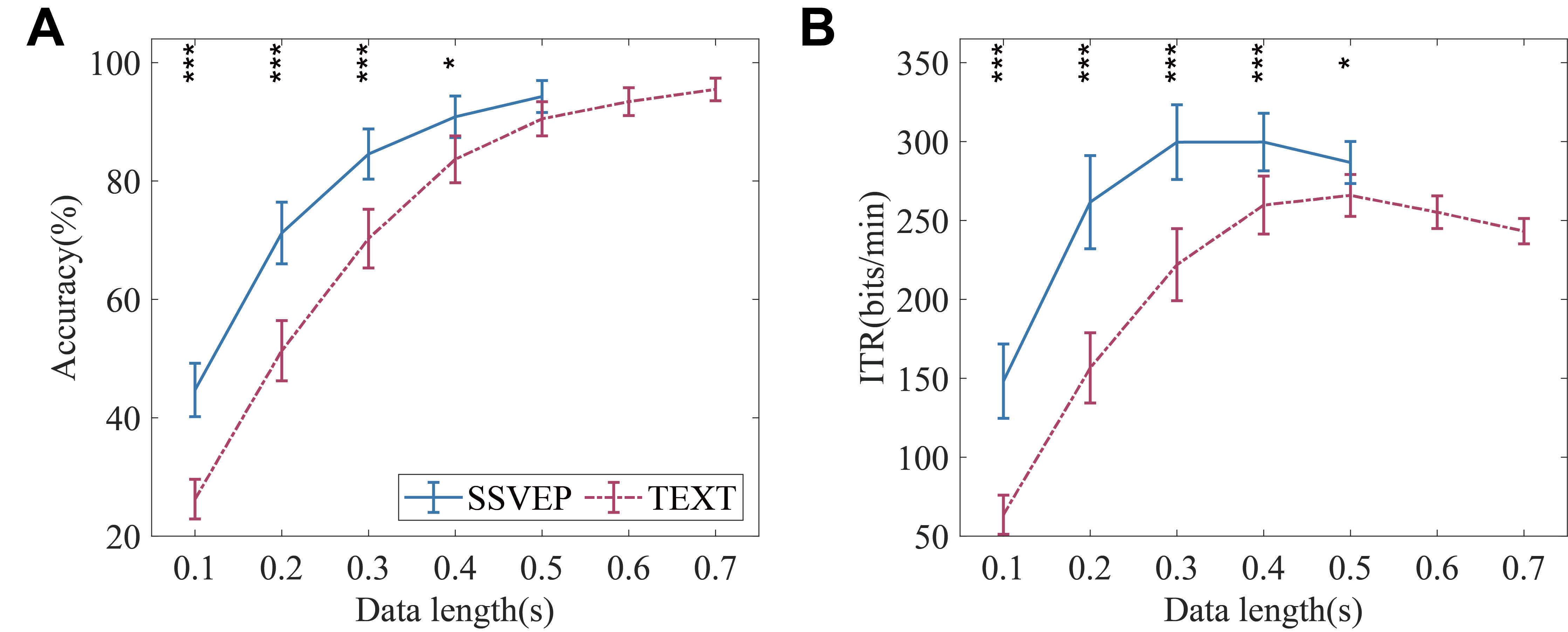
***

***Fig. S9.*** *Performance of the 40-target BCI paradigm of Text and conventional SSVEP at different time length. (A) Classification accuracy and (B) Information transfer rate (paired t-test, * p<0.05, ** p<0.01, *** p<0.001).*

**Individual differences**

In the frequency-sweep experiment of the text sequence paradigm, substantial inter-subject variability was observed. Classification accuracies ranged from 74.17% to 99.79%, with the corresponding accuracies and ITRs summarized in Table S2. Such differences are likely associated with individual variability in the temporal, spatial, and spectral properties of EEG responses. To better illustrate these inter-subject differences and their impact on classification performance, we further presented the topographical distributions, spectral responses, and transient ERPs for all participants.

***Table S2.*** *Results of the frequency-sweep experiment*

| Subject | Accuracy (%) | ITR（bits/min） |
| --- | --- | --- |
| Sub1 | 96.88 | 247.81 |
| Sub2 | 96.46 | 245.69 |
| Sub3 | 97.71 | 252.16 |
| Sub4 | 90.21 | 217.10 |
| Sub5 | 99.79 | 264.47 |
| Sub6 | 94.38 | 235.61 |
| Sub7 | 94.58 | 236.59 |
| Sub8 | 90.63 | 218.88 |
| Sub9 | 86.88 | 203.37 |
| Sub10 | 83.75 | 191.14 |
| Sub11 | 98.96 | 259.17 |
| Sub12 | 96.46 | 245.69 |
| Sub13 | 83.13 | 188.79 |
| Sub14  Sub15  Sub16  Sub17  Sub18  Sub19  Sub20 | 99.58  74.79  78.75  74.17  83.96  96.88  89.58 | 263.05  158.75  172.63  156.62  191.94  247.81  214.47 |
| Sub21 | 83.96 | 191.94 |

Fig. S10 presents the SNR topographies under 3–12 Hz stimulation across all subjects. Most participants demonstrated two distinct activation patterns across frequencies, with stronger responses typically observed in the 5–7 Hz range. However, frequency sensitivity varied across individuals: for instance, Sub1 showed enhanced responses around 8 Hz, whereas Sub8 exhibited stronger activity at higher frequencies (10–12 Hz).

Fig. S11 illustrates the relationship between stimulation frequency and SSVEP responses together with the background spectral profile. Substantial inter-subject differences were observed in harmonic response strength. For example, Subjects 13 and 15 showed relatively weak harmonic components, whereas Sub15 and Sub17 exhibited pronounced alpha-band activity across all conditions.

Fig. S12 depicts the scalp topographies of transient ERP components. Almost all participants exhibited clear P1 and N170 components over occipito-temporal regions in response to text stimuli. Nevertheless, inter-subject variability was evident in hemispheric lateralization. For instance, Sub5 showed a markedly right-lateralized N170.

Taken together, these results reveal considerable individual variability in spatial lateralization, frequency sensitivity, harmonic structure, and background neural activity. Such factors likely contribute to the observed variability in classification performance. Importantly, however, the key spatial and temporal neural signatures of the proposed paradigm were consistently observed across participants, supporting the consistency of the key neural signatures across the tested cohort

**
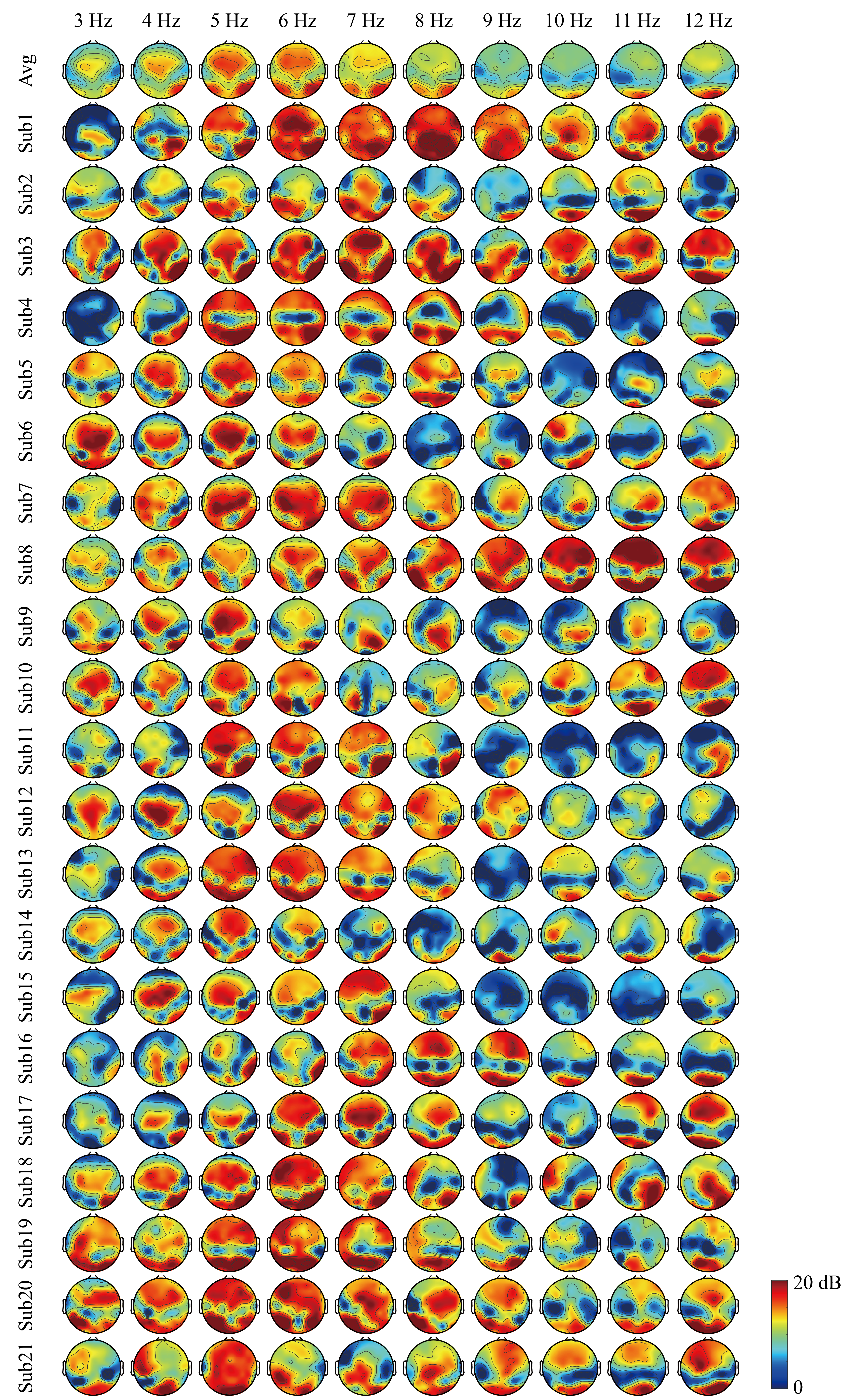
**

| ***Fig. S10.*** *Individual SNR topographies for all subjects across 3-12Hz.*  ***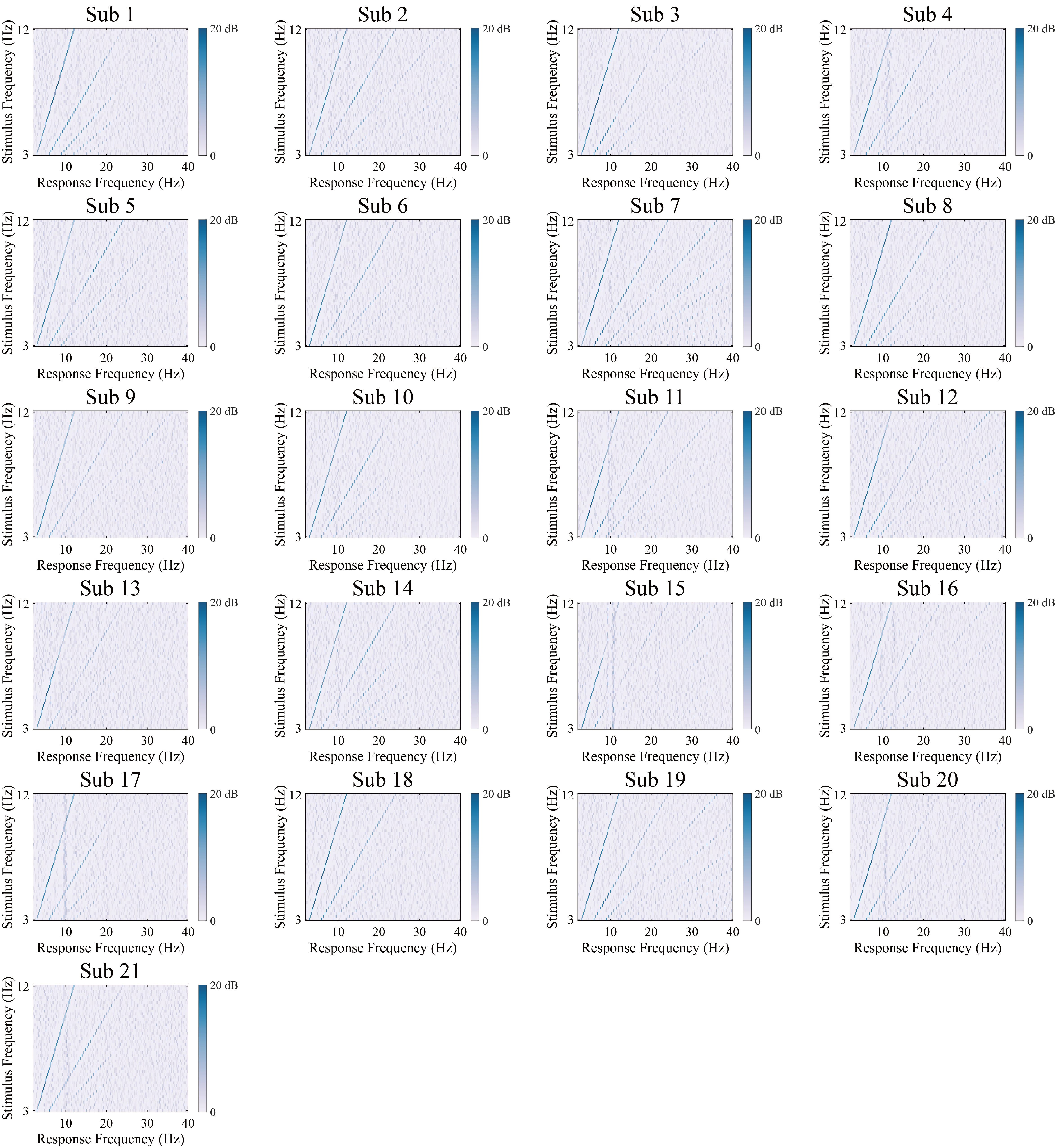***   | ***Fig. S11.*** *The relationship between stimulation frequency and EEG SNR for All subjects* | | --- |   ***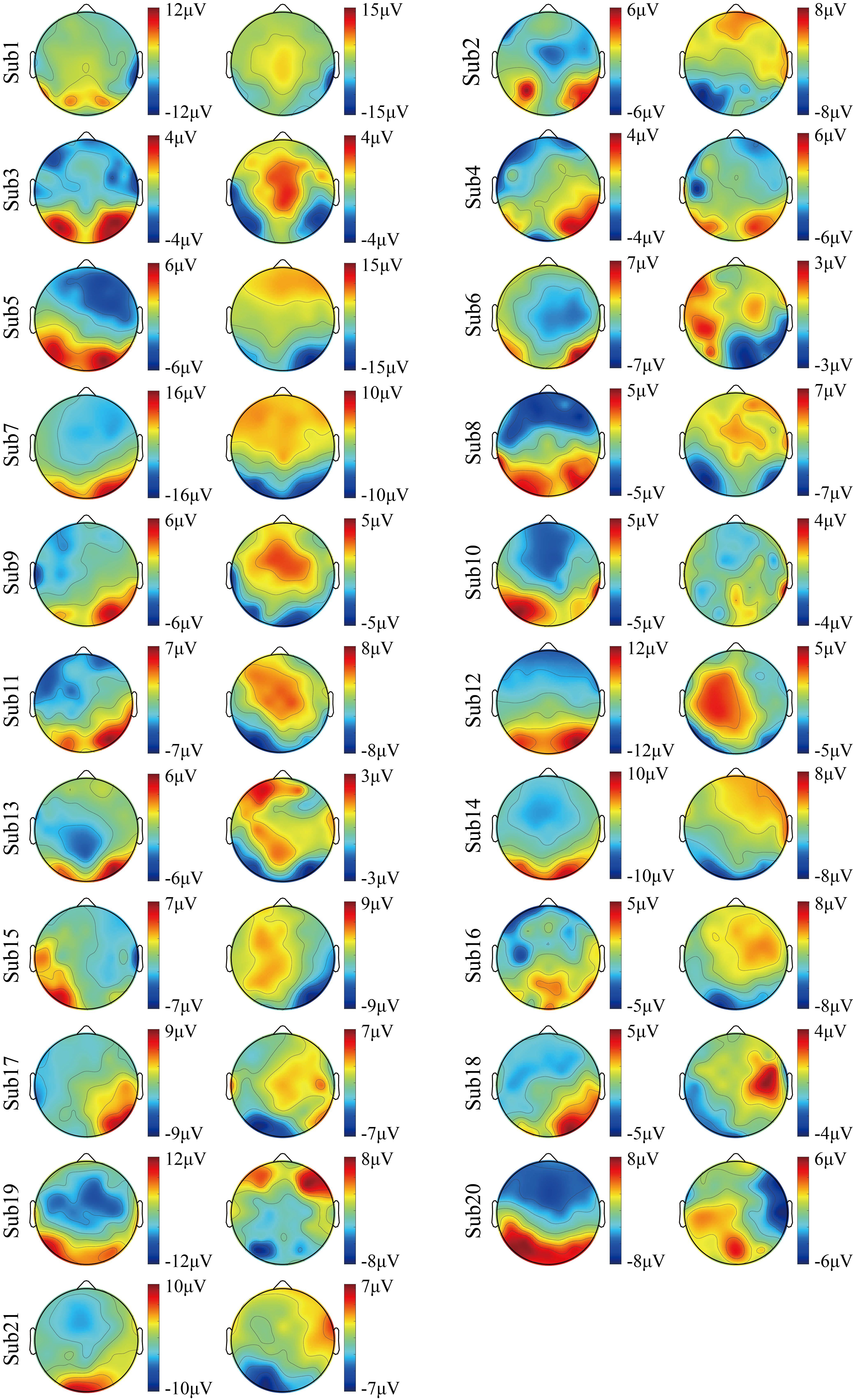***  ***Fig. S12.*** *P1 and N170 response topographies under 12Hz stimulation for All subjects.* |
| --- | --- |
